# Increased vaginal *Gardnerella vaginalis* abundance and reduced D-galactose metabolism are associated with preterm birth in older mothers with columnar ectopy in South China

**DOI:** 10.1128/msystems.00825-25

**Published:** 2025-08-15

**Authors:** Xiaohong Wang, Tingting Liang, Zhuang Liang, Tong Jiang, Ya Chen, Tong Chen, Bo Dong, Qingping Wu, Yu Gao

**Affiliations:** 1 Obstetrical Department, The Sixth Affiliated Hospital of Sun Yat-sen University, Guangzhou, Guangdong, China; 2 Key Laboratory of Human Microbiome and Chronic Diseases (Sun Yat-sen University), Ministry of Education, Guangzhou, China; 3 Biomedical Innovation Center, The Sixth Afiliated Hospital, Sun Yat-sen University, Guangzhou, China; 4 Guangdong Provincial Key Laboratory of Microbial Safety and Health, State Key Laboratory of Applied Microbiology Southern China, National Health Commission Science and Technology Innovation Platform for Nutrition and Safety of Microbial Food, Institute of Microbiology, Guangdong Academy of Sciences, Guangzhou, China; 5 Department of Rehabilitation Hospital Pain Ward, Honghui Hospital, Xi'an Jiaotong University, Xi'an, Shaanxi, China; The University of Texas at Dallas, Dallas, Texas, USA

**Keywords:** vaginal microbiome, advanced maternal age, preterm birth, metabolome, columnar ectopy

## Abstract

Advanced maternal age is associated with adverse pregnancy outcomes. However, the impact of changes in maternal physiology, biochemistry, vaginal microbiome, and its metabolites related to reproductive age on pregnancy outcomes is uncertain. Multi-omics were used to analyze vaginal samples collected from 195 pregnant women of different age groups to characterize the factors affecting the occurrence of adverse pregnancy outcomes in older mothers. Results showed that advanced maternal age is associated with a higher risk of premature birth. Furthermore, columnar ectopy may also contribute to premature birth. Moreover, older mothers with columnar ectopy exhibited vaginal microbiome dysbiosis, characterized by an increase in *Gardnerella vaginalis* and a reduction in *Lactobacillus crispatus*. Functional analysis of the microbiome revealed that disruptions in the vaginal microbiome might contribute to columnar ectopy by influencing carbohydrate metabolism in the host, such as galactose metabolism. Finally, galactose-degrading enzymes of *L. crispatus* were enriched in older mothers with non-columnar ectopies. In addition, *G. vaginalis* promoted the occurrence of columnar ectopy, whereas the dominance of galactosidase in *L. crispatus* was protective against columnar ectopy. Our findings demonstrate that *L. crispatus* is a target to prevent columnar ectopy or age-associated premature birth. *G. vaginalis* and galactose can be diagnostic markers for the occurrence of adverse pregnancy outcomes in older mothers.

**IMPORTANCE:**

Advanced maternal age poses a growing challenge to maternal health globally, yet the mechanisms linking age-related physiological changes to adverse pregnancy outcomes remain unclear. This study identifies vaginal microbiome dysbiosis, characterized by increased *Gardnerella vaginalis* and reduced *Lactobacillus crispatus*, as a critical mediator of age-associated preterm birth in mothers with columnar ectopy. By integrating multi-omics analyses, we reveal that disruptions in galactose metabolism driven by microbial shifts may contribute to columnar ectopy development. Notably, *L. crispatus* galactosidase activity emerges as a protective factor, while *G. vaginalis* and galactose accumulation serve as potential diagnostic markers. These findings provide actionable targets for microbiome-based interventions to mitigate age-related pregnancy complications, advancing personalized strategies for maternal care.

## INTRODUCTION

Age-appropriate childbearing remains a global concern. The trend of delayed marriage and childbirth has increased worldwide (1). This may be attributed to changes in social norms, increased opportunities for education and career advancement for women, and advancements in assisted reproductive techniques (2, 3). According to one report, 2.5% of women who gave birth between 2006 and 2015 were aged 15–17, 29.1% to women aged 18–24 years, 28.6% to women aged 25–29 years, 24.9% to women aged 30–34 years, and 14.9% were over 35 years old (4). Furthermore, due to the widespread implementation of the three-child policy, the age range of pregnant women in China has shifted, resulting in a gradual increase in the number of older pregnant women (5). Globally, there is an increasing trend in the number of pregnant women aged 35 years (6). Several studies have reported a correlation between maternal age and adverse pregnancy outcomes, including preeclampsia, postpartum hemorrhage, gestational diabetes, fetal miscarriage, and infant mortality (4, 7). The occurrence of adverse pregnancy outcomes, such as fetal growth restriction and low-birth-weight infants, may be attributed to various maternal factors, including obesity, gravidity, parity, use of *in vitro* fertilization-embryo transfer (IVF-ET), columnar ectopy, and scarred uterus. These factors may act either alone or in combination. Some studies have reported an association between advanced maternal age and preterm births (8, 9). This may be due to an increase in the prevalence of overweight and obesity, advanced maternal age, and growth of at-risk populations. However, the etiopathology of preterm birth remains largely unknown. Therefore, it is important to identify additional risk factors. Furthermore, there is an urgent need to identify multidimensional biomarkers to assist in the early diagnosis and therapeutic intervention of preterm birth in older mothers.

Several studies have indicated that vaginal microbiota changes with age and that age-related microbiota can lead to increased intestinal permeability and systemic inflammation, causing the loss of health-related vaginal microbiota characteristics (10, 11). Furthermore, some studies have highlighted the correlation between changes in serum physiological and biochemical indicators and vaginal microbes during pregnancy. However, no research has been conducted on the relationship among reproductive age, biochemical indicators, and vaginal microbes. Therefore, it is crucial to employ clinical, biochemical, and microbiological indicators to determine the effect of reproductive age on maternal health.

Vaginal microorganisms can serve as biomarkers to reflect maternal health status during pregnancy (12, 13). For instance, bacterial vaginosis is characterized by the depletion of *Lactobacillus* species and increased microbial diversity (14). A high diversity of vaginal microbiota, such as *Gardnerella* and *Fannyhessea*, has also been linked to bacterial vaginosis (15–17). chen et al. (2022) suggested that *Atopobium* spp. (*Fannyhessea*) may play a significant role in the pathogenesis of spontaneous abortion (18). The composition of the maternal vaginal microbiome has been linked to preterm births (19). Specifically, *Lactobacillus crispatus* is often associated with a lower risk of preterm births (20). Disturbances in the vaginal microbiome have been linked to adverse pregnancy outcomes. However, previous studies on the association between the vaginal microbiome and preterm birth have reported contradictory results. Sherrianne et al. reported that the composition of the vaginal microbiota was not associated with the risk of preterm birth in pregnant African-American women. This may be due to the low preterm birth rates observed, as well as the differences in biogeography and ethnicity (21). Our investigation revealed that whereas clinical indicators such as fetal fibronectin (22) and cervical length (17) have been identified as potential biomarkers for preterm birth, few stable and reliable biomarkers of preterm birth have been discovered. This is due to either low specificity or insufficient evidence. Therefore, it is necessary to clarify the relationship between preterm birth and vaginal microbiome during pregnancy in the Chinese population, including its influencing factors, particularly in older mothers. Furthermore, our understanding of the specific mechanisms underlying potential host-microbiome interactions in preterm births is limited.

Several studies have reported that changes in vaginal microbiota and metabolites are associated with the development and regulation of pregnancy-related diseases. Ziklo et al. (23) reported that dysbiosis of the vaginal microbiota and a higher vaginal kynurenine/tryptophan ratio were associated with Chlamydia trachomatis genital infections (23). Kindschuh et al. (2021) reported multiple associations between vaginal metabolites, such as diethanolamine and ethylglucoside, and subsequent preterm births (24). Flavia et al. (25) identified a composite of metabolites (leucine, tyrosine, aspartate, lactate, betaine, acetate, and Ca^2+^) associated with the risk of preterm birth (25). These studies demonstrated the potential of vaginal metabolites as early biomarkers of adverse pregnancy outcomes. Further studies are needed to understand how vaginal microbes and their metabolite signatures interact in older mothers. Further research is required to investigate the impact of the vaginal ecosystem on prematurity and other adverse pregnancy outcomes in older mothers.

Thus, in the present study, to identify non-invasive and efficient biomarkers for adverse pregnancy outcomes in older mothers, we recruited pregnant women and systemically measured, analyzed, and compared their vaginal biochemical parameters, via high-throughput sequencing of 16S rDNA, combined with metagenomic sequencing and LC-MS-based vaginal metabolites. Machine-learning algorithms were then developed to predict subsequent preterm births in older mothers with columnar ectopy, based on the vaginal microbiome and metabolomes. Our results demonstrate a promising approach for studying the potential causes of prematurity and early risk stratification in older mothers and highlight the need to study high-risk factors for preterm birth.

## RESULTS

### Basic characteristics of the study cohort

**Fig 1:**
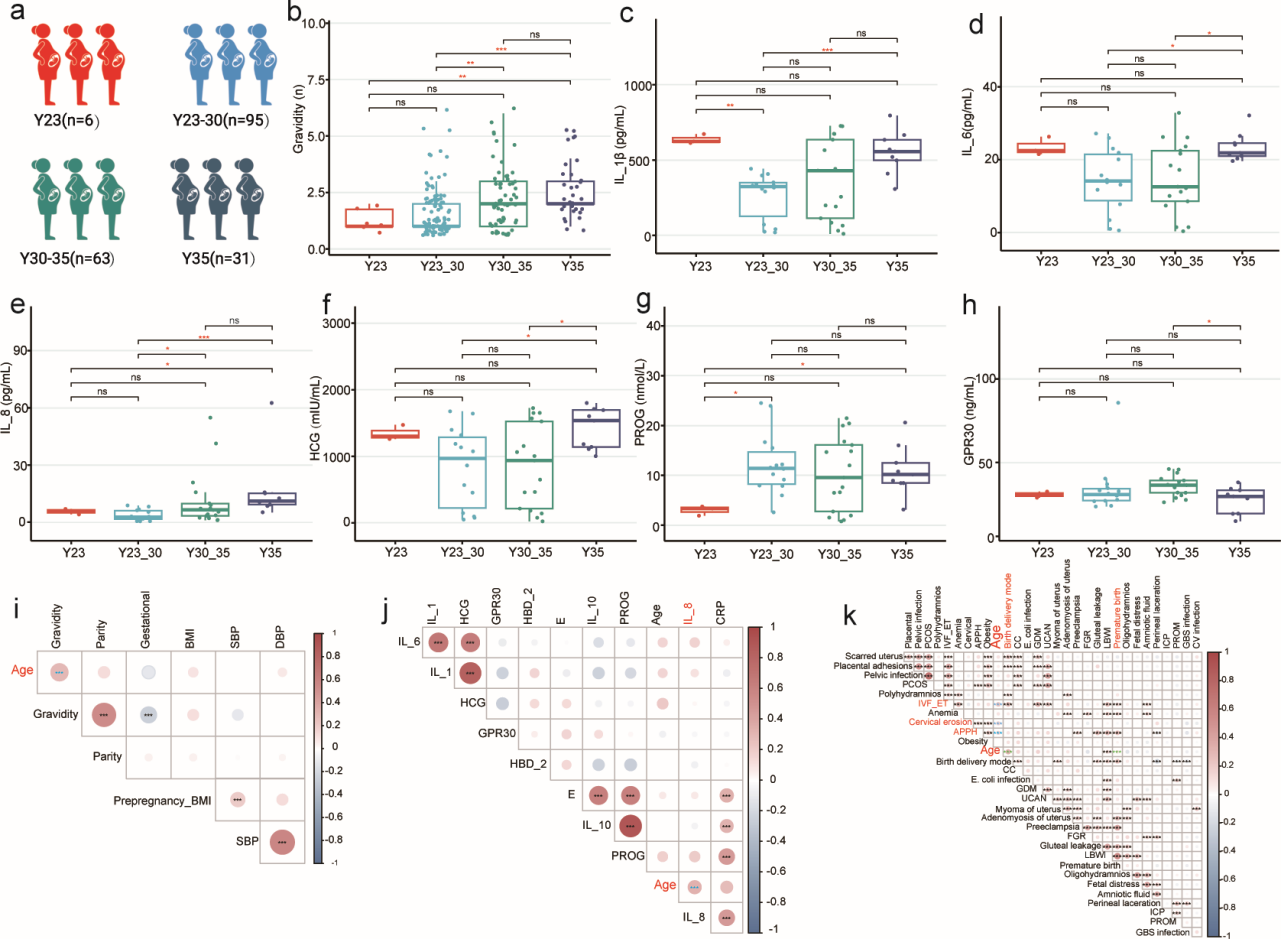
Clinical characteristics and biochemical indicators among parturients of different reproductive ages. (**a**) Study group. (**b**) Gravidity. (**c**) IL-1β. (**d**) IL-6. (**e**) IL-8. (**f**) HCG. (**g**) PROG. (**h**) GPR30. (**i**) Heatmap of Spearman correlations between clinical index. (**j**) Heatmap of Spearman correlations between clinical index and biochemical indicators. (**k**) Heatmap of Spearman correlations between clinical index and other clinical outcomes. IL-1β, interleukin-1β; IL-6, interleukin-6; IL-8, interleukin-8; HCG, human chorionic gonadotropin; PROG, progesterone; GPR30, G protein-coupled estrogen receptor 30; FGR, fetal growth restriction; LBWI, low birth weight infants; CC, cervical cerclage; IVF-ET, *in vitro* fertilization-embryo transfer; APPH, adverse pregnancy production history; PCOS, polycystic ovary syndrome; ICP, intrahepatic cholestasis of pregnancy; GDM, gestational diabetes mellitus; GBS, *Streptococcus agalactiae*; CV, *Candida vulvovaginal; E. coli, Escherichia coli*; PROM, premature rupture of membranes; UCAN, umbilical cord around neck. *R* values represent positive (red) or negative (blue) correlations. ^*^P < 0.05, ^**^P < 0.01, ^***^P < 0.001.

A total of 195 participants were recruited for the study. The study population was classified into four groups based on maternal age to explore the association between maternal factors, vaginal microbes, and pregnancy outcomes in women of different ages. According to the Baidu Baike (https://baike.baidu.com/item/Best reproductive age/546225), the age range of 23–30 is the optimal age for women to conceive, thus <23-year-old group, 23- to 30-year-old group, 30- to 35-year-old group, and >35-year-old group at a mean gestation of 23 weeks were included in this study (Fig. 1a). Table 1 presents the maternal factors for the four age groups, including height, weight, BMI, gestational age, gravidity, parity, SBP, and DBP. Women in the Y35 group had significantly higher gravidity than those in the Y23 group (*P <* 0.05) (Fig. 1b). However, there were no significant differences in the other clinical characteristics among the four groups (*P >* 0.05) (Fig. S1a through d). Table 1 shows that maternal age was a significant factor for pregnancy outcomes. Compared to mothers aged 23–35 years, teenage mothers aged 17–22 years, and older mothers aged 36–41 years have an increased risk of adverse pregnancy outcomes, including fetal growth restriction, low birth weight infants, and premature birth. This study recorded the use of assisted reproductive technology, including cervical and *in vitro* fertilization-embryo transfer (IVF-ET), as well as adverse pregnancy history. Among the groups studied, the Y35 group had the highest rate of IVF-ET use (25.8%) (Table 1). Table 1 presents the occurrence of pregnancy comorbidities such as columnar ectopy, scarred uterus, uterine myoma, uterine adenomyosis, amniotic fluid, oligohydramnios, polyhydramnios, pelvic infection, PCOS, ICP, GDM, obesity, preeclampsia, anemia, GBS, CV, *E. coli* infection, and PROM. The incidence of columnar ectopy in pregnant women significantly increased with age, particularly in the Y30-35 and Y35 groups, reaching 23.8% and 22.6%, respectively. However, the incidence of GBS infection in the Y23-30 group (14.7%) was significantly higher than that in the other three groups (*P <* 0.05), and further studies are required. Table 1 shows the statistical analysis of parturition complications in pregnant women, including fetal distress, perineal laceration, gluteal leakage, UCAN, placental adhesions, and birth delivery mode (cesarian section, vaginal birth, or abortion). Compared with mothers aged 23–30 years, teenage mothers aged 17–22 years, and older mothers aged 36–41 years had a significantly higher rate of cesarian delivery, reaching 50.0% and 71.0%, respectively. Pregnant women aged 23–30 and 30–35 showed a trend toward vaginal delivery. Furthermore, older mothers had a higher incidence of abortions.

**TABLE 1:** Detailed clinical characteristics in pregnant women of the recruited subjects^*a*^

Variables	Y23 (*N* = 6)	Y23–30 (*N* = 95)	Y30–35 (*N* = 63)	Y35 (*N* = 31)	*χ*2	*P*
Clinical index						
Sample proportion	3.0%	48.7%	32.3%	16.0%	–^*b*^	
Age	21 (17–22)	28 (24–30)	33 (31–35)	38 (36–41)	–	<0.001
Height (cm)	157.5 (150–160)	159.2 (147–173)	158.5 (148–175)	157.9 (148–167)	–	0.554
Prepregnancy weight (kg)	61.9 (47.0–75.0)	56.1 (30.0–82.0)	55.2 (42.5–75.5)	58.4 (42.5–84.0)	–	0.230
Prepregnancy BMI	24.9 (20.3–31.5)	22.1 (12.7–30.1)	21.9 (17.3–28.8)	23.4 (18.1–33.7)	–	0.066
Gravidity (*n*)	1 (1–2)	2 (1–6)	2 (1–6)	3 (1–5)	–	<0.001
Parity (*n*)	0 (0–1)	0 (0–2)	1 (0–3)	1 (0–2)	–	0.282
SBP (mmHg)	112.5 (94.0–130.0)	110.0 (85.0–150.0)	109.8 (85.0–140.0)	111.1 (92.0–148.0)	–	0.909
DBP (mmHg)	65.8 (50.0–80.0)	67.4 (51.0–94.0)	67.5 (47.0–95.0)	68.7 (57.0–82.0)	–	0.807
Occurrence of adverse pregnancy outcomes						
FGR (*n*)	0 (0)	4 (4.2%)	5 (8.0%)	4 (12.9%)	6.799	0.079
LBWI (*n*)	0 (0)	4 (4.2%)	5 (8.0%)	0 (0)	11.396	0.070
Premature birth (*n*)	1 (16.7%)	5 (5.3%)	4 (6.3%)	6 (19.4%)	7.067	0.010
Adverse pregnant production history and assisted reproductive technology (ART)						
CC (*n*)	0 (0)	4 (4.2%)	5 (8.0%)	4 (12.9%)	3.451	0.327
IVF-ET (*n*)	0 (0)	4 (4.2%)	8 (12.7%)	8 (25.8%)	13.010	0.005
APPH (*n*)	0 (0)	13 (13.7%)	13 (20.6%)	9 (29.0%)	5.380	0.146
Occurrence of pregnancy comorbidities						
Cervical erosion (*n*)	1 (16.7%)	8 (8.4%)	15 (23.8%)	7 (22.6%)	7.960	0.047
Scarred uterus (*n*)	1 (16.7%)	8 (8.4%)	8 (12.7%)	5 (16.1%)	1.805	0.614
Myoma of uterus (*n*)	0 (0)	4 (4.2%)	3 (4.8%)	1 (3.2%)	0.390	0.942
Adenomyosis of uterus (*n*)	0 (0)	2 (2.1%)	2 (3.2%)	2 (6.5%)	1.677	0.642
Amniotic fluid (*n*)	1 (16.7%)	5 (5.3%)	3 (4.8%)	1 (3.2%)	1.893	0.595
Oligohydramnios (*n*)	1 (16.7%)	2 (2.1%)	1 (1.6%)	0 (0)	7.097	0.069
Polyhydramnios (*n*)	1 (16.7%)	2 (2.1%)	1 (1.6%)	1 (3.2%)	5.151	0.161
Pelvic infection (*n*)	0 (0)	1 (1.1%)	4 (6.3%)	0 (0)	5.455	0.141
PCOS (*n*)	0 (0)	1 (1.1%)	5 (7.9%)	0 (0)	7.469	0.058
ICP (*n*)	0 (0)	2 (2.1%)	0 (0)	1 (3.2%)	1.862	0.601
GDM (*n*)	1 (16.7%)	8 (8.4%)	10 (15.9%)	8 (25.8%)	6.318	0.097
Obesity (*n*)	3 (50.0%)	11 (11.6%)	8 (12.7%)	6 (19.4%)	7.565	0.056
Preeclampsia (*n*)	0 (0)	2 (2.1%)	0 (0)	1 (3.2%)	1.862	0.601
Anemia (*n*)	0(0)	7 (7.4%)	6 (9.5%)	6 (19.4%)	4.517	0.211
GBS infection (*n*)	0 (0)	14 (14.7%)	3 (4.8%)	0 (0)	9.097	0.028
CV infection (*n*)	1 (16.7%)	11 (11.6%)	4 (6.3%)	2 (6.5%)	1.931	0.587
*E. coli* infection (*n*)	0 (0)	0 (0)	3 (4.8%)	0 (0)	6.384	0.094
PROM (*n*)	0 (0)	20 (21.1%)	10 (15.9%)	2 (6.5%)	4.925	0.177
Occurrence of parturition complications						
Fetal distress (*n*)	1 (16.7%)	3 (3.2%)	1 (1.6%)	0 (0)	6.623	0.085
Perineal laceration (*n*)	1 (16.7%)	8 (8.4%)	6 (9.5%)	1 (3.2%)	1.742	0.628
Gluteal leakage (*n*)	0 (0)	3 (3.2%)	1 (1.6%)	1 (3.2%)	0.587	0.899
UCAN (*n*)	0 (0)	4 (4.2%)	4 (6.3%)	2 (6.5%)	0.793	0.851
Placental adhesions (*n*)	0 (0)	2 (2.1%)	4 (6.3%)	1 (3.2%)	2.226	0.527
Birth delivery mode						
Cesarean (*n*)	3 (50.0%)	22 (23.2%)	28 (44.4%)	22 (71.0%)	30.428	<0.001
Born vaginally (*n*)	3 (50.0%)	72 (75.8%)	34 (54.0%)	7 (22.6%)		
Abortion (*n*)	0 (0)	1 (1.1%)	1 (1.6%)	2 (6.5%)		

^*a*^ FGR, fetal growth restriction; LBWI, low birth weight infants; CC, cervical cerclage; IVF-ET, *in vitro* fertilization-embryo transfer; APPH, adverse pregnant production history; PCOS, polycystic ovary syndrome; ICP, intrahepatic cholestasis of pregnancy; GDM, gestational diabetes mellitus; GBS, *Streptococcus agalactiae*; CV, *Candida vulvovaginal; E. coli, Escherichia coli*; PROM, premature rupture of membranes; UCAN, umbilical cord around neck.

^*b*^ "-" represents the absence of chi square test.

Biochemical indicators of pregnant women in different age groups were detected, including IL-1β, IL-6, IL-8, IL-10, HCG, PROG, E, HBD-2, GPR30, and CPR. The indicators were then grouped based on the four age groups for statistical analysis. Compared to mothers aged 23–30 years, teenage mothers aged 17–22 years, and older mothers aged 36–41 years exhibited higher levels of IL-1β (*P <* 0.05) (Fig. 1c). Furthermore, the Y35 group had higher IL-6 levels than the Y23–30 and Y30–35 groups (*P <* 0.05) (Fig. 1d). IL-8 and HCG levels showed a significantly increasing trend with age (*P <* 0.05) (Fig. 1e and f). The results indicated that Y35 and Y23–30 had a higher level of PROG than teenage mothers aged 17–22 years (*P <* 0.05) (Fig. 1g). Moreover, the level of GPR30 showed a trend of first increasing and then decreasing, with the Y30–35 group having the highest level of GPR30 compared to Y35 (*P <* 0.05) (Fig. 1h). However, there were no significant differences in serum IL-10, E, HBD-2, or CRP levels among the four groups (Fig. S1e through h).

To investigate the correlation between maternal age and clinical laboratory examinations, biochemical indicators, and other maternal factors, a heatmap was created using the Spearman correlation analysis. Maternal age was positively correlated with gravidity (Fig. 1i) and IL-8 levels (Fig. 1j), as well as with birth delivery mode, premature birth, IVF-ET, APPH, and columnar ectopy (Fig. 1k). These findings indicate that maternal age has a significant effect on these indicators, including adverse outcomes.

### Vaginal microbiome taxonomic indicators of pregnant women vary with maternal age using 16S rRNA gene sequencing

**Fig 2:**
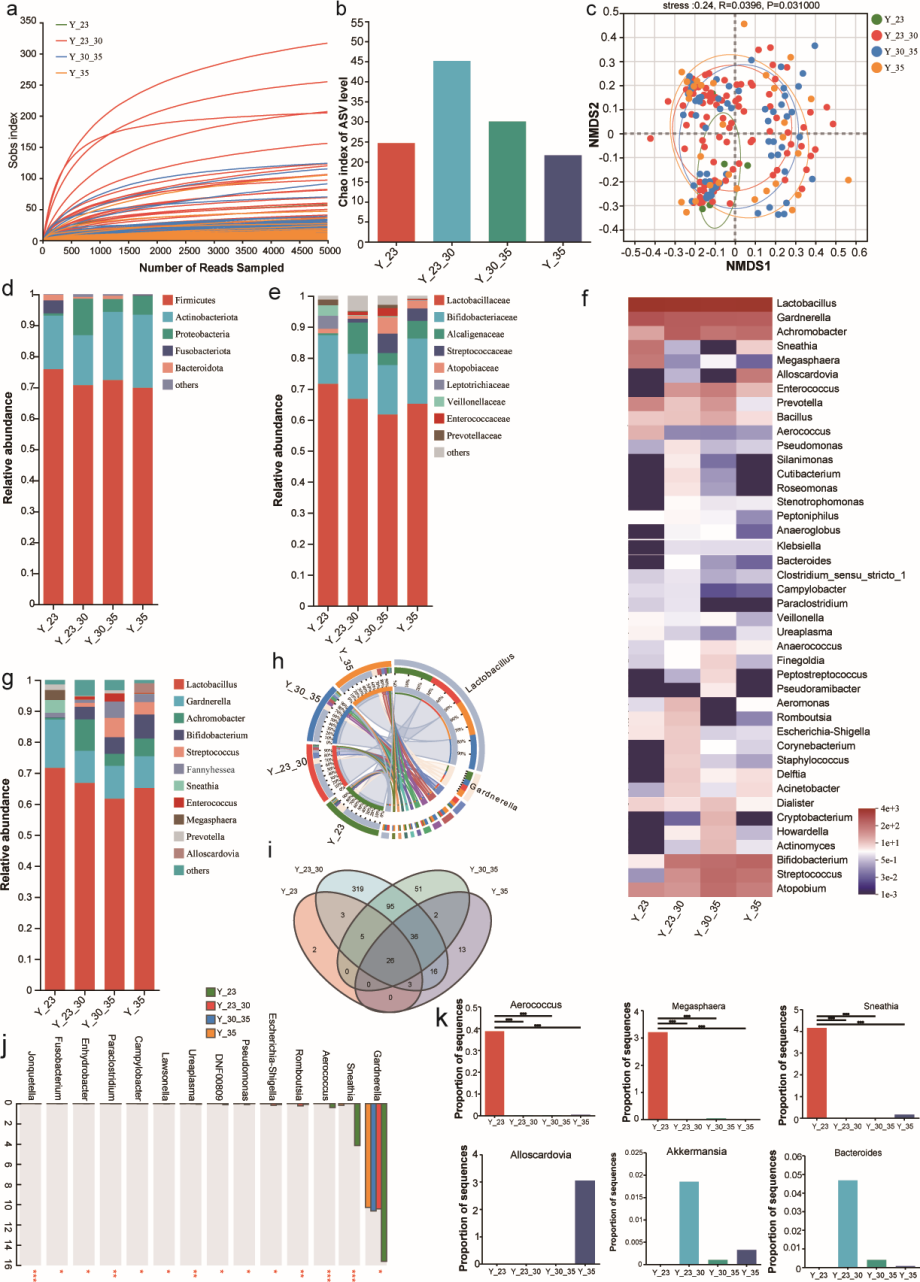
Alpha-diversity, beta-diversity, and composition of vaginal microbial community among parturients of different reproductive ages. (**a**) Sobs curve for each sample. (**b**) The microbial diversity estimated by Chao index. (**c**) NMDS analysis of the bacterial community in parturients of different reproductive ages at the ASV level. (**d**) Relative abundance of the microbial community at the phylum level in parturients of different reproductive ages. (**e**) Relative abundance of the microbial community at the family level in parturients of different reproductive ages. (**f**) Heatmap of the microbial community at the genus level in parturients of different reproductive ages. (**g**) Relative abundance of the microbial community at the genus level in parturients of different reproductive ages. (**h**) Circos of the microbial community at the genus level in parturients of different reproductive ages. (**i**) Venn diagrams of the parturients of different reproductive ages according to microbial biodiversity at the genus level. (**j**) Histogram of the vaginal microbiota at the genus level. (**k**) Comparison of vaginal microbial composition in parturients of different reproductive ages at the genus level. ^*^P<0.05, ^**^P<0.01, ^***^P<0.001.

A total of 16,960,487 high-quality reads, with an average length of 424.98 bp, were obtained from 195 vaginal discharge samples. The Shannon and Sobs curves reached steady plateaus (Fig. S2a; Fig. 2a), indicating a sufficient sequencing depth for analyzing most bacterial communities. Meanwhile, the Chao, Ace, and Sobs indices, which represent community richness and diversity, decreased in the other three groups when compared to the Y23–30 group, particularly in the Y35 group. This suggests a difference in the bacterial biodiversity between the Y23–30 and Y35 groups. However, no significant differences were observed between the two groups (Fig. 2b; Fig. S2c and d). To investigate whether there was a difference in the vaginal microbiota composition of mothers aged 36–41 compared to mothers of other ages, we performed hierarchical clustering (unweighted FastUnifrac) and NMDS analyses (Fig. 2c; Fig. S2d). The bacterial communities were classified into four types at the ASV level based on the composition of the bacterial community in all samples, indicating a clear difference in the vaginal microbiota among different maternal ages.

A relative abundance analysis was conducted for 33 phyla, 281 families, and 571 genera across all samples. Figure S3a through e shows that 1,122,16, and 6 families were obtained from samples in the Y23, Y23–30, Y30–35, and Y35 groups, respectively. Among these, 23 families were common in all samples (Fig. S3f). Furthermore, the Y23–30 sample had the highest number of unique families (122).

Figure 2 displays the relative abundance of vaginal bacterial communities at the phylum, family, and genus levels. Firmicutes had the most phylogroups detected in the samples, followed by Actinobacteria, which is consistent with prior investigations of bacterial biodiversity in America (26). This difference may be due to differences in regions and races. In teenage mothers aged 17–22 years, Fusobacteria was the dominant phylum, whereas Proteobacteria were more abundant in the Y23–30, Y30–35, and Y35 groups. Furthermore, the relative abundance of Bacteroidetes decreased with increasing age, with the Y35 group showing the lowest abundance of Bacteroidetes (Fig. 2d). At the family level, most samples were abundant in *Lactobacillaceae* and *Bifidobacteriaceae*, which is consistent with previous reports (21). Compared with the other three groups, the Y23–30 group had the highest number of *Alcaligenaceae*. The Y23 group had unique families of *Leptotrichiaceae* and *Veillonellaceae*. The family *Enterococcaceae* was abundant in the Y30–35 group, and *Streptococcaceae* and *Atopobiaceae* were predominant in the Y30–35 and Y35 groups (Fig. 2e). At the genus level, *Lactobacillus* and *Gardnerella* were enriched in all the samples (Fig. 2f through h). The Y23–30 group had the highest number of unique genera, reaching 319 (Fig. 2i). LDA Effect Size (LEfSe) was used to identify the key vaginal microbes that were associated with maternal age. As shown in Fig. 2j and k, the other three groups had a lower relative abundance of *Gardnerella* and a higher relative abundance of *Achromobacter*, *Bifidobacterium*, and *Streptococcus* than teenage mothers aged 17–22 years. Furthermore, the Y23 group exhibited higher levels of *Aerococcus*, *Sneathia*, and *Megasphaera*, whereas the Y30–35 and Y35 groups had greater abundances of *Fannyhessea* and *Alloscardovia* than the Y23–30 group. In contrast, the potential probiotics *Akkermansia* and *Bacteroides* were decreased in the Y30–35 and Y35 groups. These findings suggest a potential association between age-related changes in physical function and the abundance of vaginal microbes.

### Association of vaginal microbial species with clinical traits

**Fig 3:**
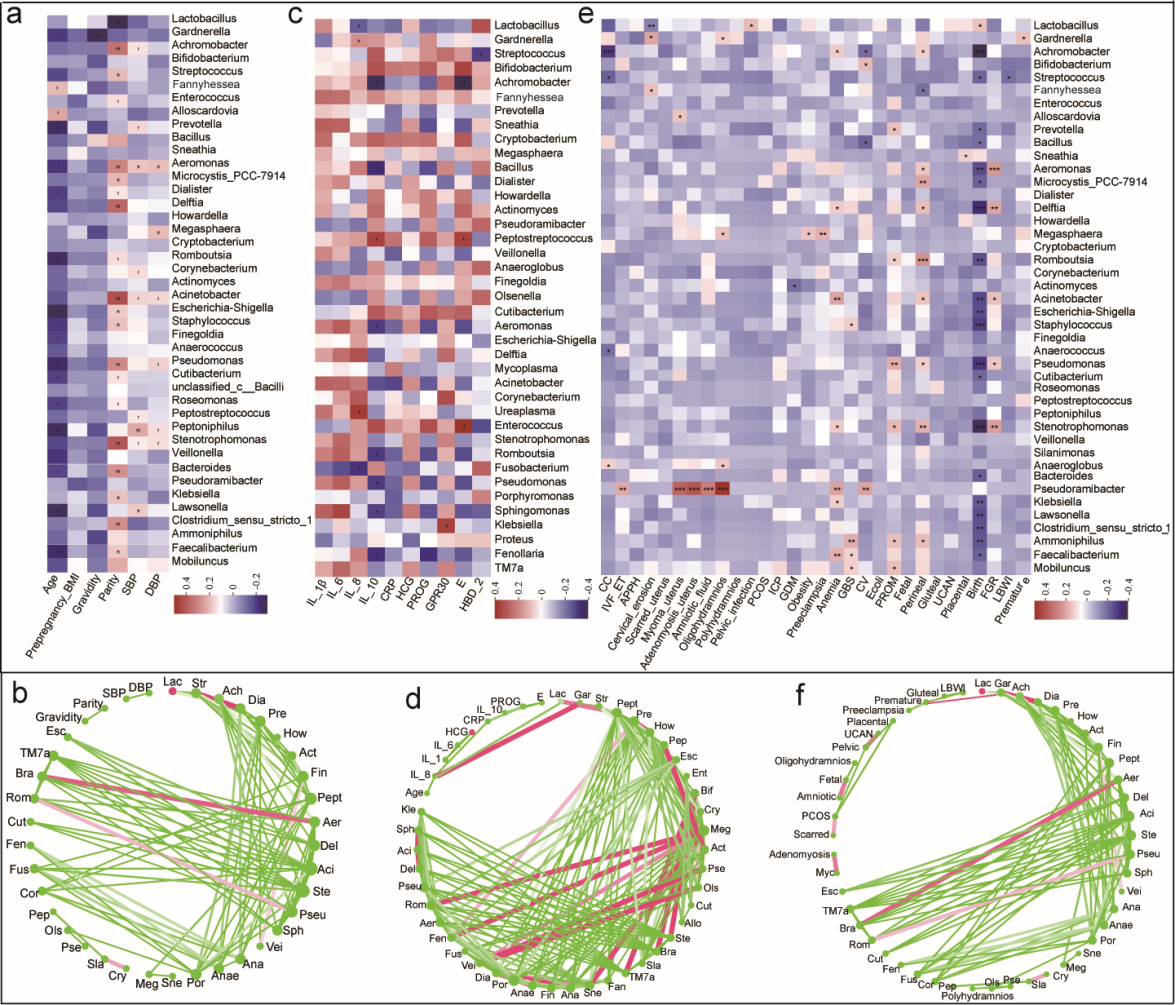
Correlational analyses between clinical indicators, biochemical indicators, occurrence of parturition complications, and composition of vaginal microbial community among parturients of different reproductive ages. (**a**) Heatmap of Spearman correlations between vaginal microbiota and clinical indicators at the genus level. (**b**) A co-occurrence network between vaginal microbiota and clinical indicators at the genus level. (**c**) Heatmap ofSpearman correlations between vaginal microbiota and biochemical characteristics at the genus level. (**d**) A co-occurrence network between vaginal microbiota and biochemical characteristics at the genus level. (**e**) Heatmap of Spearman correlations between vaginal microbiota and occurrence of parturition complications at the genus level. (**f**) A co-occurrence network between vaginal microbiota and occurence of parturition complications at the genus level. The *R* value is shown in different colors in the fig. The color indicates a positive (red) or negative (blue) correlation. ^*^P < 0.05, ^**^P < 0.01, ^***^P < 0.001.

Spearman’s correlation tests were conducted to investigate the relationship between vaginal microbial species and clinical indicators related to maternal age. The results indicated a significant association between maternal vaginal microbes and age-related maternal clinical and biochemical indicators (Fig. 3). The abundance of *Lactobacillus* was negatively correlated with mothers' age and parity. The abundance of certain genera, including *Aeromonas*, *Acinetobacter*, *Escherichia-Shigella*, and *Staphylococcus*, increased in teenage mothers aged 17–22 years, and was positively correlated with mothers’ age and parity. Furthermore, *Gardnerella* was negatively correlated with age and gravidity. In contrast, older mothers aged 36–41 years had an increased abundance of *Fannyhessea* and *Alloscardovia*, which were positively correlated with age. *Fannyhessea* was also positively correlated with gestational age (Fig. 3a and b).

Correlation analysis was performed between the taxa and host biochemical indicators. IL-8, which was increased in mothers aged 36–41 years, was negatively correlated with *Lactobacillus* and *Fusobacterium*, whereas it exhibited positive correlations with *Gardnerella* and *Ureaplasma. Achromobacter*, *Bacillus*, *Aeromonas*, *Romboutsia*, *Pseudomonas*, *Sphingomonas*, and *Fenollaria* were enriched in teenage mothers aged 17–22 years and were negatively correlated with IL-10. The levels of IL-10 were positively correlated with *Peptostreptococcus*, whereas PROG was negatively correlated with *Fenollaria*. GPR30 was positively correlated with *Klebsiella*, whereas *E. Streptococcus* was negatively correlated with *Achromobacter*. However, *Enterococcus* was positively correlated with *Peptostreptococcus* spp. In contrast, HBD_2 negatively correlated with *Streptococcus* (Fig. 3c and d).

This study revealed that *Lactobacillus* was negatively correlated with cervical erosion, whereas *Gardnerella* and *Fannyhessea* were positively correlated. Furthermore, *Gardnerella* was positively correlated with premature birth. This study also found that *Achromobacter*, *Streptococcus*, and *Anaerococcus* were negatively correlated with CC, whereas Anaeroglobus was positively correlated with CC. *Alloscardovia* increased in older mothers aged 36–41 years and was positively correlated with myoma_uterus. *Sneathia*, *Aeromonas*, and *Megasphaera* were enriched in teenage mothers aged 17–22 years and were positively correlated with placental adhesions, FGR, oligohydramnios, obesity, and preeclampsia. Furthermore, *Pseudoramibacter* positively correlated with IVF_ET. This study found a positive correlation between *Lactobacillus* and birth delivery mode. However, several other bacteria, including *Achromobacter*, *Streptococcus*, *Prevotella*, *Bacillus*, *Aeromonas*, *Delftia*, *Romboutsia*, *Acinetobacter*, *Escherichia-Shigella*, *Staphylococcus*, *Pseudomonas*, and *Stenotrophomonas*, were negatively correlated with birth delivery mode (Fig. 3e and f).

The data suggest a potential link between the vaginal microbiome, specifically *Lactobacillus* and *Gardnerella*, and adverse pregnancy outcomes, such as premature birth in mothers aged 36–41. This link may be related to maternal factors, such as gravidity, IVF-ET, birth delivery mode, and cervical erosion.

### Increasing *Gardnerella vaginalis* and decreasing *Lactobacillus crispatus* induce the occurrence of cervical erosion may be the main maternal factor of influencing the premature birth

Mothers aged 36–41 years were divided into two groups based on gravidity. Figure S4 shows that G3-5 had lower Chao and Shannon indices than G1-2, but the difference was not significant (*P >* 0.05) (Fig. S4a and b). The beta diversity analysis of the vaginal microbiome was similar in G1-2 and G3-5 (*P >* 0.05) (Fig. S4c). At the phylum level, the abundance of Firmicutes, Actinobacteria, and Proteobacteria was higher in women in G1-2, whereas Actinobacteria and Firmicutes were the two main phyla in G3-5 (Fig. S4d). When comparing the vaginal microbial composition at the genus level between the two groups, we observed large differences in the abundance of the genera *Lactobacillus* and *Gardnerella*, but these differences were not statistically significant (*P <* 0.05) (Fig. S4e through g). The genera *Streptococcus*, *Alloscardovia*, *Fannyhessea*, *Sneathia*, *Klebsiella*, and *Achromobacter* did not show significant differences (*P <* 0.05). These results suggest that gravidity may not be the primary factor contributing to premature births in pregnant women.

Mothers aged 36–41 years were divided into two groups based on whether IVF-ET was used. IVF_ET had lower Chao and Shannon indices than N_IVF_ET, but no significant difference was observed between the two groups (*P >* 0.05) (Fig. S5a and b). The beta diversity analysis of the vaginal microbiome was similar between the N_IVF_ET and IVF_ET groups (*P >* 0.05) (Fig. S5c). At the phylum level, all samples exhibited abundant Firmicutes, Actinobacteria, and Proteobacteria (Fig. S5d). The vaginal microbial composition at the genus level was compared between the two groups. The abundance of the genera *Lactobacillus*, *Achromobacter*, *Anaerococcus*, *Prevotella*, and TM7x was higher in N_IVF_ET than in IVF_ET, whereas *Gardnerella*, *Alloscardovia*, and *Klebsiella* were more abundant in IVF_ET than in N_IVF_ET. However, there were no significant differences between the groups (*P <* 0.05), as shown in Fig. S5e through g. These results suggest that IVF may not be the primary maternal factor contributing to premature birth in pregnant women.

All mothers aged 36–41 years were divided into two groups based on birth delivery mode. As shown in Fig. S6, alpha and beta diversity analyses of the vaginal microbiome were similar between the two groups (*P* > 0.05) (Fig. S6a through c). Born vaginally exhibited more abundant Firmicutes, Actinobacteria, Proteobacteria, and Firmicutes, and Actinobacteria constituted the two main phyla in the cesarian group (Fig. S6d). We compared the vaginal microbial composition at the genus level between the two groups. The abundance of the genera *Lactobacillus*, *Achromobacter*, *Gardnerella, Fannyhessea*, *Sneathia*, and *Escherichia-Shigella* was not significantly different between the two groups (*P* > 0.05) (Fig. S6e through g). These results indicate that delivery mode may not be the main maternal factor affecting premature birth in pregnant women.

**Fig 4:**
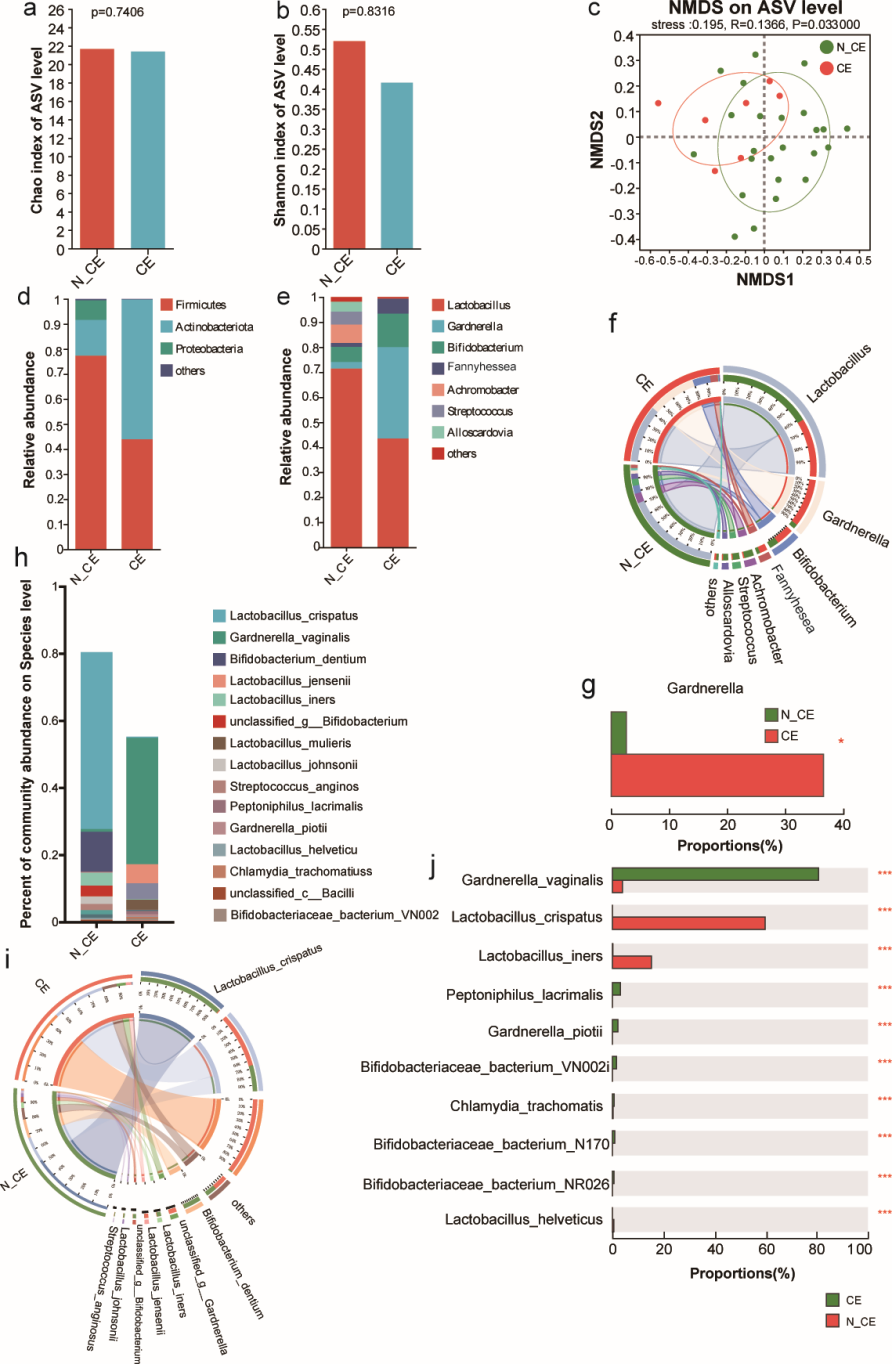
Alpha-diversity, beta-diversity, and composition of vaginal microbial community in the older mothers with columnar ectopy and non_columnar ectopy. (**a**) The microbial diversity estimated by Chao index. (**b**) The microbial diversity estimated by Shannon index. (**c**) NMDS analysis of the bacterial community in the older mothers with columnar ectopy at the ASV level. (**d**) Relative abundance of the microbial community at the phylum level in the older mothers with columnar ectopy and non-columnar ectopy. (**e**) Relative abundance of the microbial community at the genus level in the older mothers with columnar ectopy and non-columnar ectopy. (**f**) Circos of the microbial community at the genus level in the older mothers with columnar ectopy and non-columnar ectopy. (**g**) Comparison of the relative abundance of *Gardnerella* in the older mothers with columnar ectopy and non-columnar ectopy and non-columnar ectopy. (**h**) Relative abundance of the microbial community at the species level in the older mothers with columnar ectopy and non-columnar ectopy. (**i**) Circos of the microbial community at the species level in the older mothers with columnar ectopy and non-columnar ectopy. (**j**) Histogram of the vaginal microbiota at the species level in the older mothers with columnar ectopy and non-columnar ectopy. ^*^P < 0.05, ^**^P < 0.01, ^***^P < 0.001.

Mothers aged 36–41 were divided into two groups based on the presence or absence of columnar ectopy. Figure 4 shows that columnar ectopy (CE) had lower Chao and Shannon indices than N_CE, but the difference was not significant (*P >* 0.05) (Fig. 4a and b). The beta diversity results for the vaginal microbiome were significantly different between the two groups (*P <* 0.05) (Fig. 4c). N_CE exhibited a higher abundance of Firmicutes, Actinobacteria, and Proteobacteria, with Actinobacteria being the most abundant phylum in the CE group (Fig. 4d). At the genus level, there were no significant differences in the abundance of *Lactobacillus*, *Streptococcus*, *Achromobacter*, *Fannyhessea*, *Prevotella*, or *Bifidobacterium* between the two groups (*P >* 0.05) (Fig. S7a), but the genus *Gardnerella* was significantly more abundant in the CE group than in the N_CE group (*P <* 0.05) (Fig. 4e through g).

Metagenomic sequencing was performed to investigate the relationship between specific species of *Gardnerella* and CE. This study found that *L. crispatus*, *Bifidobacterium dentium*, and *L.* iners were significantly lower in the CE group than in the N_CE group. In contrast, *the abundance of G. vaginalis* was markedly higher in the CE group than in the N_CE group (Fig. 4h through j; Fig. S7b). These results suggest that *G. vaginalis* may induce CE, which could be a significant maternal factor contributing to premature births in pregnant women. Furthermore, *L. crispatus* is considered a functional probiotic that inhibits the growth of *G. vaginalis*.

### Functional characterization of the pregnant women’s vaginal microbiome with CE

**Fig 5:**
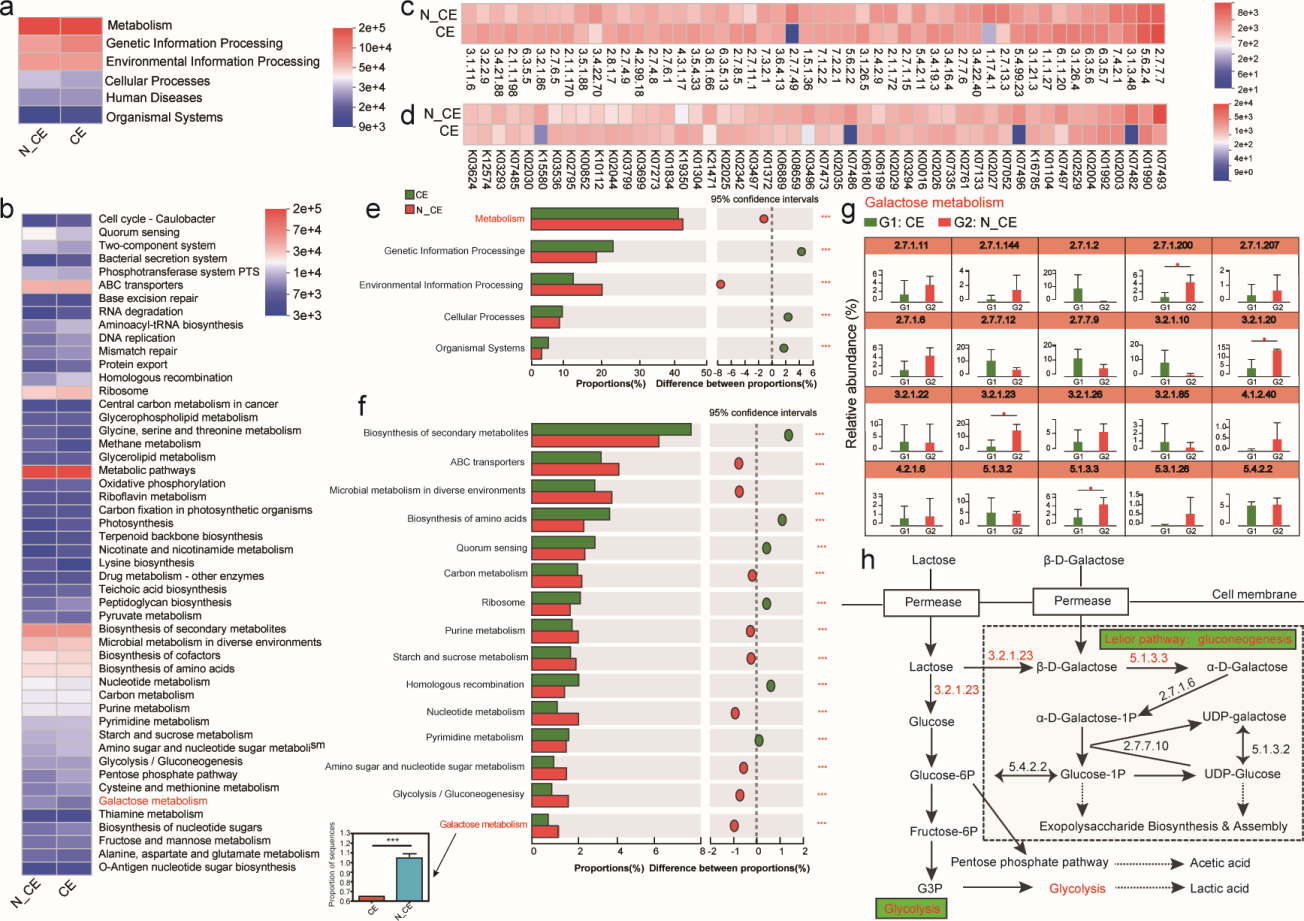
The functions of the bacterial community in the older mothers with columnar ectopy and non-columnar ectopy. (**a**) Overall KEGG gene function statistics (Level 1). (**b**) The heatmap of functional pathways (Level 3). (**c**) The enzymes in the older mothers with columnar ectopy and non-columnar ectopy. (**d**) The pathways in the older mothers with columnar ectopy and non-columnar ectopy. (**e**) Histogram of functional composition at the level 1. (**f**) Histogram of functional composition at the level 3. (**g**) Comparison of the relative abundance of enzymes related to galactose metabolism (ko00052) pathways in the older mothers with columnar ectopy and non-columnar ectopy. (**h**) The key enzymes of 2.7.1.200, 3.2.1.20, 3.2.1.23, and 5.1.33 in pathways of galactose metabolism, involved in the classical Lelior pathway: gluconeogenesis, were significantly higher enriched in the vaginal microbiome of the older mothers in N_CE individuals than in CE samples.

KEGG analysis was used to identify the biological functions of each bacterial species (Fig. 5a and e). The analysis revealed six domains abundant in older mothers in the CE and N_CE groups: metabolism, genetic information processing, environmental information processing, cellular processes, human diseases, and organism systems. The enrichment of metabolism-related genes in all samples suggested that metabolism plays a key role in the vaginal microbiome. The KEGG pathways related to the analysis of “Metabolic pathways,” “Biosynthesis of secondary metabolites,” and “Microbial metabolism in diverse environments” were more prevalent in both groups. Furthermore, “galactose metabolism,” which participates in carbohydrate metabolism, was more enriched in the vaginal microbiome of older mothers in the N_CE group than in that of the CE group (Fig. 5b and f). Enzymes related to galactose metabolism (ko00052) pathways were annotated, including 6-phosphofructokinase (2.7.1.11), tagatose-6-phosphate kinase (2.7.1.144), glucokinase (2.7.1.2), galactitol PTS permease (2.7.1.200), lactose PTS permease (2.7.1.207), galactokinase (2.7.1.6), UDP-glucose---hexose-1-phosphate uridylyltransferase (2.7.7.12), UDP glucose pyrophosphorylase (2.7.7.9), oligo-1,6-glucosidase (3.2.1.10), alpha-glucosidase (3.2.1.20), alpha-galactosidase (3.2.1.22), beta-galactosidase (3.2.1.23), beta-fructofuranosidase (3.2.1.26), 6-phospho-beta-galactosidase (3.2.1.85), tagatose-bisphosphate aldolase (4.1.2.40), galactonate dehydratase (4.2.1.6), UDP-glucose 4-epimerase (5.1.3.2), galactose mutarotase (5.1.3.3), galactose-6-phosphate isomerase (5.3.1.26), and phosphoglucomutase (5.4.2.2). Among them, two key enzymes (beta-galactosidase 3.2.1.23 and galactose mutarotase 5.1.3.3) involved in the classical Leloir pathway of galactose metabolism and gluconeogenesis were significantly more enriched in the vaginal microbiome of older mothers in N_CE individuals than in CE samples (Fig. 5c, d, g and h; Fig. S8).

**Fig 6:**
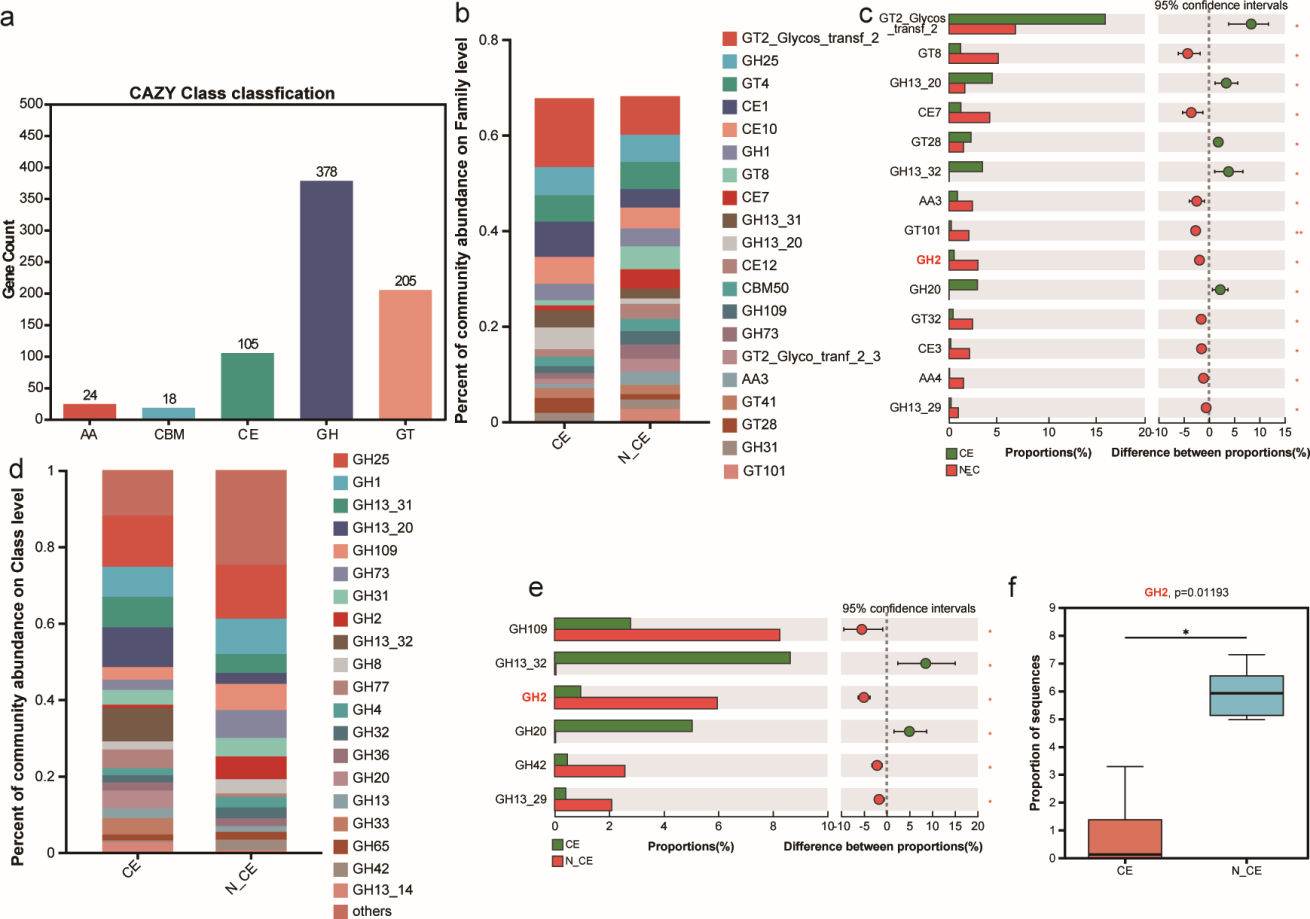
CAZyme annotation profile of the older mothers with columnar ectopy and non-columnar ectopy. (**a**) Overview of CAZyems classes in total the older mothers with columnar ectopy and non-columnar ectopy microbes. (**b**) Relative abundance of CAZymes subfamilies in the older mothers with columnar ectopy and non-columnar ectopy. (**c**) Histogram of CAZymes subfamilies in the older mothers with columnar ectopy and non-columnar ectopy. (**d**) Relative abundance of GH subfamily members in the older mothers with columnar ectopy and non-columnar ectopy. (**e**) GH subfamily members in the older mothers with columnar ectopy and non-columnar ectopy. (**f**) Comparison of the relative abundance of GH2 in the older mothers with columnar ectopy and non-columnar ectopy. CAZyme, carbohydrate-active enzyme; GH, glycoside hydrolases; GT, glycosyl transferases; AA, auxiliary activities;CBM, carbohydrate-binding modules; CE, carbohydrate esterases.

This study aimed to investigate the effect of CE on vaginal microbial enzymes involved in carbohydrate metabolism in older mothers. Gene families in the CAZy database were searched for scaffolds (Fig. 6). The related gene counts of GHs (378) and GTs (205) were enriched in all samples at the CAZY Class classification level, followed by carbohydrate esterase CEs (105), auxiliary activities (AAs; 24), and carbohydrate-binding modules (CBMs; 18). The family level is shown in Fig. 6b and c. The abundance of GT8, CE7, AA3, GT101, GH2, GT32, CE3, AA4, and GH13_29 was higher in N_CE samples than in CE samples (*P <* 0.05). Conversely, GT2_Glycos_transf_2, GH13_20, GT28, GH13_32, and GH20 were significantly lower in N_CE samples than in CE samples (*P <* 0.05). To investigate the effect of glycoside hydrolases on the galactose metabolism pathway, we analyzed the GH levels of GH109, GH2, GH42, and GH13_29. Our results showed these enzymes were significantly more abundant in N_CE samples than in CE samples (*P <* 0.05). The GH2 genes involved in the galactose metabolism pathway were significantly upregulated in the N_CE vaginal microbiome compared to those in the CE samples (*P <* 0.05) (Fig. 6d and f).

### Effects of *Lactobacillus crispatus* on galactose metabolism

The dominance of *L. crispatus* in vaginal microbiota is associated with vaginal health (27). Therefore, we analyzed the potential relationship between *L. crispatus* in the vaginal microbiota and galactose metabolism. The “galactose metabolism” pathway, which is present in vaginal commensals such as *L. crispatus* from the *Lactobacillus* genus, was observed to possess highly specific glycoside hydrolases for metabolizing host lactose and galactose. GH36, GH2, GH32, GH31, and GH13_31 enzymes were depleted in the vaginal microbiome of CE individuals compared to N_CE controls (Data S1). Microorganisms can metabolize D-galactose through two pathways: the Leloir and De Ley-Doudoroff pathways (DD)(28). Furthermore, it was discovered that N_CE subjects were enriched in *L. crispatus*, which contained genes encoding galactonate dehydratase [EC:4.2.1.6] (K01684, converting D-galactonate to glycerate-3P; see Data S1) via the DD pathway. We found that *L. crispatus*, which also contained genes encoding UDP-glucose 4-epimerase [EC: 5.1.3.2] (K01784, converting galactose to alpha-D-glucose-1P; Data S1) and aldose 1-epimerase [EC: 5.1.3.3] (K01785, converting galactose to alpha-D-glucose-1P; Data S1) via the Leloir pathway was depleted in CE subjects. These results suggest that an increase in *L. crispatus* may contribute to the lower circulating concentrations of galactose in subjects with CE.

**Fig 7:**
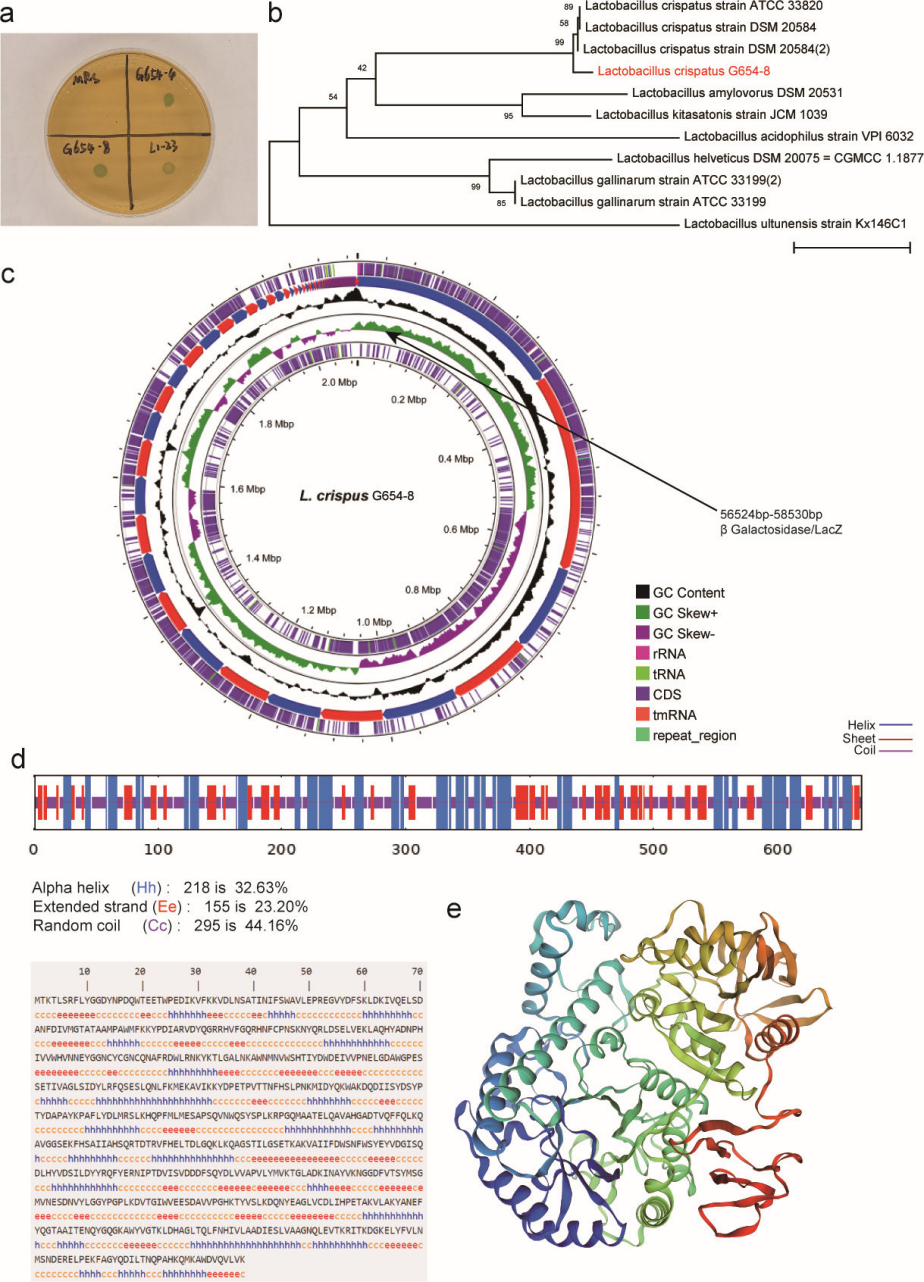
Galactose metabolism by *Lactobacillus crispatus* species may modulate the levels of circulating the lactose and galactose that are correlated with the occurrence of columnar ectopy in older mothers. (**a**) strains of *Lactobacillus crispatus* (G654-4, G654-8, and L1-23), isolate from vaginal secretions of healthy pregnant women, co-culturing with Xgal *in vitro*. (**b**) The construction of the phylogenetic trees of the *Lactobacillus crispatus* G654-4 and other genus of *Lactobacillus*. (**c**) The gene *LacZ*, encoding beta-galactosidase in *Lactobacillus crispatus* G654-4. (**d**) The secondary structure of beta-galactosidase protein in *Lactobacillus crispatus* G654-4 based on the GOR4 algorithm. (**e**) The tertiary structures of beta-galactosidase protein in *Lactobacillus crispatus* G654-4 were constructed using SWISS-MODEL.

Three strains of *L. crispatus* (G654-4, G654-8, and L1-23) were isolated from vaginal secretions of healthy pregnant women. *In vitro* co-culture with Xgal resulted in a distinct blue color, particularly with the strain *L. crispatus* G654-8. This indicated that *L. crispatus* contains beta-galactosidase in its genome (Fig. 7a and b). Figure 7b displays the evolutionary relationship between *L. crispatus* G654-4 and other species of the genus *Lactobacillus*, clarify that G654-4 belongs to the *L. crispatus* species, rather than other *lactobacilli* such as *L. iners* or *L. acidophilus*, to ensure the accuracy of strain identity in subsequent functional experiments. Combined with the X-gal staining results in Fig. 7a, it suggests that *L. crispatus* may specifically express this enzyme, while other *lactobacilli* may have different functions. The *LacZ* gene, which encodes beta-galactosidase, was annotated in the *L. crispatus* G654-4 genome (Fig. 7c), *LacZ* localization demonstrates that beta-galactosidase is an endogenous gene expressed in the strain, rather than being carried by exogenous plasmids or experimentally contaminated, providing molecular biology evidence for subsequent enzyme activity validation (such as X-gal colorimetric experiments). The GOR4 algorithm was used to predict the secondary structure of beta-galactosidase. The results indicate that the secondary structure of the protein primarily comprises an alpha helix, extended strand, and random coil, accounting for 32.63%, 23.20%, and 44.16%, respectively (Fig. 7d). Tertiary structures of the beta-galactosidase protein were constructed using SWISS-MODEL, indicating the presence of multiple alpha helices (Fig. 7e). Structural biology data is a key link connecting genes (*lacZ*) and functions (galactose metabolism), the structural characteristics of beta-galactosidase, such as the proportion of alpha helices, extended chains, and random coils, are directly related to its catalytic function. For example, the stability of alpha helices may affect the binding efficiency of enzymes with substrates such as D-galactose. The three-dimensional structural modeling further reveals the active site and spatial conformation of the enzyme, supporting the mechanistic hypothesis that *L. crispatus* exerts a protective effect by degrading galactose, such as metabolizing galactose through the Leloir pathway or De Ley Doudoroff pathway. Therefore, these findings suggest that galactose metabolism by *L. crispatus* affects circulating lactose and galactose levels, which are associated with CE in older mothers.

### Associations of vaginal microbial species, metabolites, and clinical indicators

**Fig 8:**
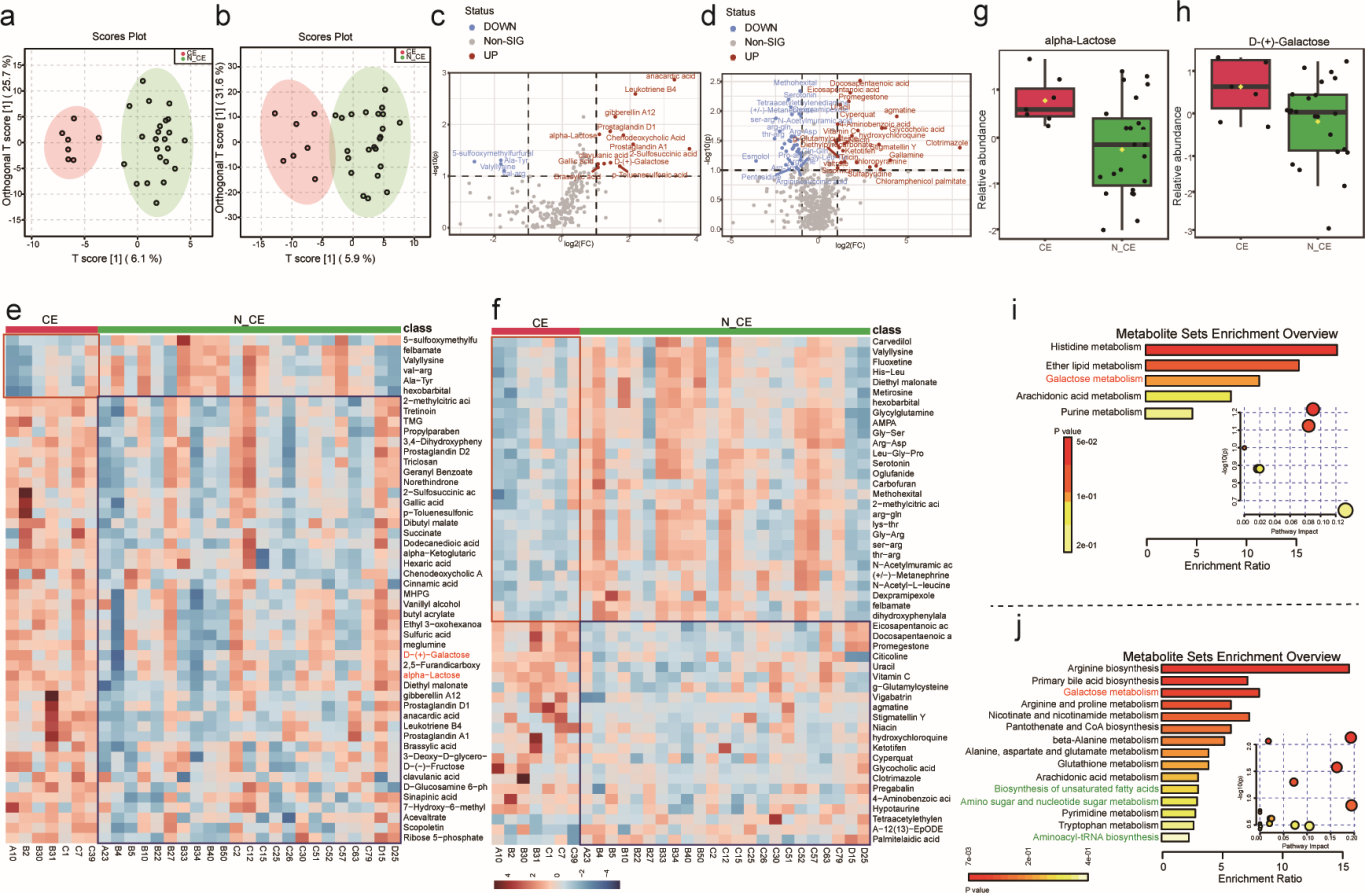
The differences of vaginal metabolites and metabolic pathway enrichment analysis between N_CE and CE groups. (**a, b**) Score plot of the OPLS-DA model constructed in the positive and negative ion modes. (**c, d**) Volcano plot was used to differentiate the metabolite signatures between N_CE and CE groups in the positive and negative ion modes. (**e, f**) Heat map showing the abundance of the differential metabolites for each sample in N_CE vs CE in the positive and negative ion modes. (**g, h**) Comparative analysis of the level of alpha-lactose and D-Galactose in different groups. (**i, j**) KEGG pathway enrichment pathway analysis of differential metabolites in N_CE vs CE in the positive and negative ion modes.

To investigate the association between the altered vaginal microbiome in subjects with CE and metabolites in older mothers, we conducted non-targeted metabolomic profiling of vaginal samples from the CE and N_CE groups. The OPLS-DA plot revealed significant differences in the vaginal metabolites of the CE group compared to those of the N_CE group (Fig. 8a and b), indicating considerable differences in the metabolites of the vaginal microbiomes of subjects with CE. This study identified 75 potential biomarkers that differentiated the CE and N_CE groups using a volcano plot. The criteria for selection were an adjusted *P*-value of less than 0.05 and a |log2(FC)|>1. The CE group upregulated 37 metabolites and downregulated 38 metabolites compared with the N_CE group (Fig. 8c through f). The concentrations of lactose and D-Galactose in the vagina differed significantly between the CE and N_CE control subjects (Fig. 8g and h).

Metabolites were classified based on their functional metabolic pathways using a metabolic pathway enrichment analysis. The main pathways associated with CE in the neg model were histidine, ether lipid, galactose, arachidonic acid, and purine metabolism (Fig. 8i). The POS model revealed that CE affected several pathways, including arginine biosynthesis, primary bile acid biosynthesis, galactose metabolism, and arginine and proline metabolism (Fig. 8j).

**Fig 9:**
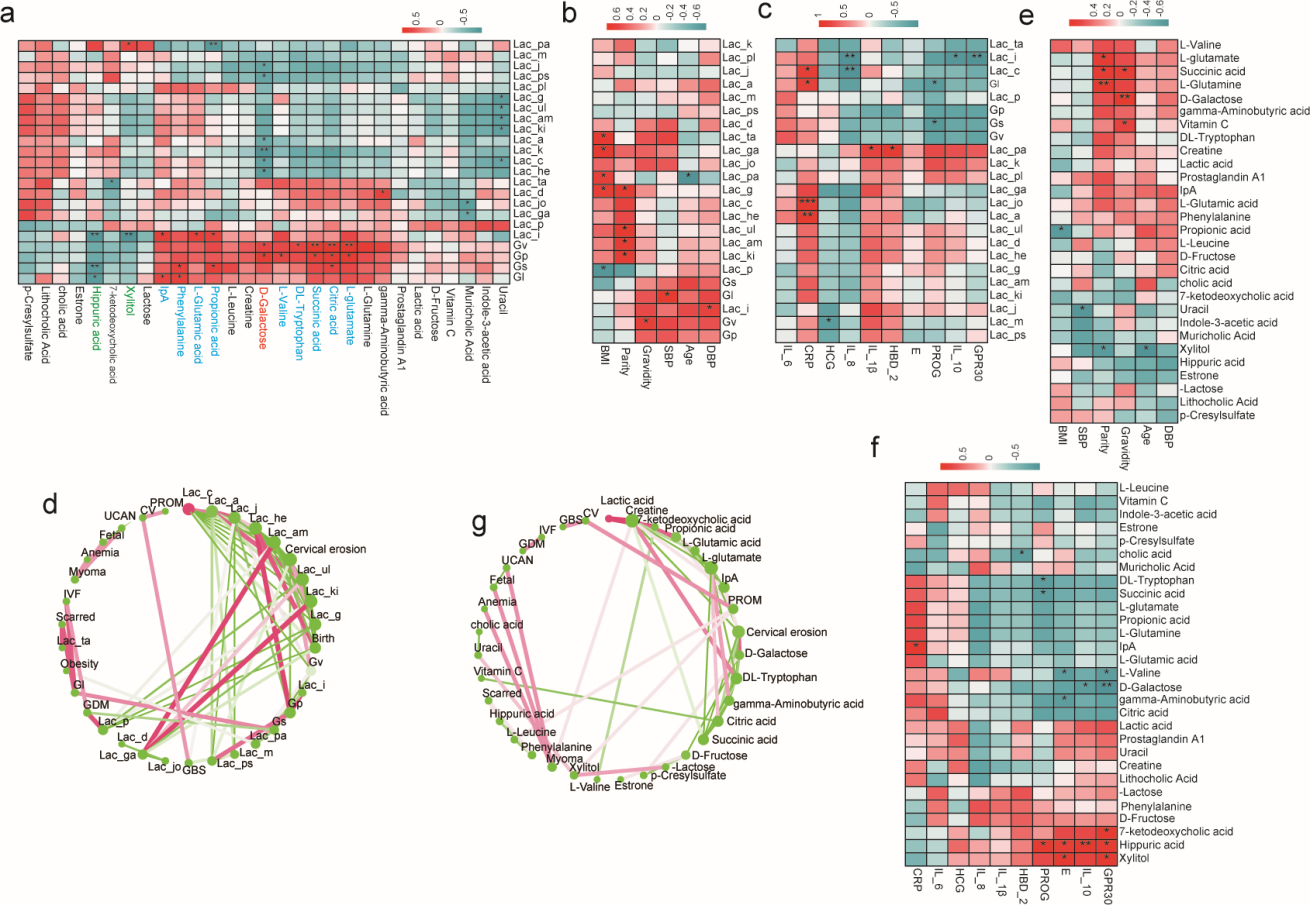
Correlation analysis among the relationship between clinical index, biochemical indicators with the vaginal microbial, and metabolites. (**a**) Heatmap of Spearman correlations between vaginal microbiota and metabolites at the species level. (**b**) Heatmap of Spearman correlations between vaginal microbiota and clinical indicators at the species level. (**c**) Heatmap of Spearman correlations between vaginal microbiota and biochemical indicators at the species level. (**d**) A co-occurrence network between vaginal microbiota and occurrence of pregnancy comorbidities at the genus level. (**e**) Heatmap of Spearman correlations between vaginal metabolites and clinical indicators. (**f**) Heatmap of Spearman correlations between vaginal metabolites and biochemical indicators. (**g**) A co-occurrence network between vaginal metabolites and occurrence of pregnancy comorbidities at the genus level. (**h**) The Sankey network of integrative statistical correlation analysis.

To investigate whether the altered abundance of metabolites correlated with the altered vaginal microbiota, Spearman’s correlation analysis was used to determine the relationship between the 23 species and 29 metabolites that differed in abundance between the CE and N_CE groups (Fig. 9a). There was a positive correlation between CE-enriched D-galactose and *Gardnerella* species such as *G. vaginalis* and *G. piotii*. Conversely, D-galactose is inversely correlated with *Lactobacillus* species, including *L. crispatus*, *L. jensenii*, *L. psittaci*, *L. acidophilus*, *L. kefiranofaciens*, and *L. helveticus*. Furthermore, certain organic acids, including citric acid, succinic acid, propionic acid, and L-glutamic acid, as well as some amino acids, such as DL-Tryptophan, L-Valine, and Phenylalanine, were correlated with *Gardnerella* species, including *G. vaginalis*, *G. piotii*, *G. leopoldii*, and *G. swidsinskii*. Xylitol and hippuric acid levels were negatively correlated with *Gardnerella* species.

This study evaluated the relationships between clinical indices, biochemical indicators, vaginal microbes, and metabolites. The results showed a positive correlation between *G. vaginalis*, D-galactose concentrations, and gravidity (Fig. 9b and e). Furthermore, *L. crispatus-enriched* N_CE samples were inversely correlated with IL-8 levels (Fig. 9c), and CE-enriched D-galactose was inversely correlated with IL-10 and GPR30 levels (Fig. 9f). Moreover, we observed a co-occurrence network between the vaginal microbes and pregnancy-related complications. Specifically, we found a negative correlation between *L. crispatus* and *G. vaginalis*, whereas *G. vaginalis* and D-galactose showed a positive correlation with the proportion of columnar ectopies (Fig. 9d and g).

Taken together, these results indicate that the altered vaginal microbiota, specifically, the reduction of the abundance of *Lactobacillus* species and the increase in that of *Gardnerella* species in subjects with CE, may be associated with higher concentrations of D-galactose and the induction of adverse pregnancy outcomes in older mothers.

## DISCUSSION

According to one report, approximately 15 million preterm infants are born globally each year (29). Adverse pregnancy outcomes were significantly affected by the maternal age (30). However, previous studies have reported contradictory findings regarding the relationship between maternal age and adverse pregnancy outcomes. Both extremes of reproductive age are considered associated with an increased risk of adverse outcomes (30, 31). A recent analysis of a large cohort found that maternal age >40 years was associated with preterm birth (9, 32, 33). However, the mechanism underlying preterm birth remains unknown, and its prevention and early risk stratification remain unclear.

To investigate this, we conducted integrative 16S rRNA gene sequencing and metagenomic and metabolomic analyses of 195 vaginal samples collected during parturition. Thirty-one samples from pregnant women of advanced maternal age and 6 samples from spontaneous preterm births in pregnant women of advanced maternal age at a mean gestation of 23 weeks were included, the incidence of premature birth is as high as 19.35%. Multiple associations were observed between maternal factors and subsequent preterm births. These factors include gravidity, IVF-ET, delivery mode, and the inflammatory IL-8. Furthermore, in our cohort, associations between the vaginal microbiome and CE were observed. *G. vaginlis* was associated with CE, suggesting that this organism may induce CE. Future studies in established model systems are warranted to determine the role of *G. vaginalis* in CE and preterm birth. This finding aligns with previous research on interactions between the vaginal microbiota and local immunity trigger inflammation, mediating spontaneous preterm birth, characterized by cervical dilatation (34). However, only seven samples from CE in pregnant women of advanced maternal age were included in this study; large-scale cohort studies are necessary for a more comprehensive understanding. Moreover, *L. crispatus* contains genes that encode glycoside hydrolases (GH2), which aid in the breakdown of galactose. This bacterium is a potentially functional probiotic that inhibits the growth of *Gardnerella vaginalis*. In addition, a strong correlation was observed between the metabolome and microbiome profiles, specifically the enrichment of *Gardnerella vaginalis* and D-galactose in CE subjects. The sources of lactose may be due to the dual contributions of host secretion and microbial metabolism, the host cervical mucus itself contains carbohydrates such as lactose, which can be normally broken down into galactose and glucose by lactobacilli (such as *L. crispatus*) through beta-galactosidase (*LacZ*). *L. crispatus* completely metabolizes galactose as an energy substance through the Leloir pathway and De Ley Doudoroff pathway, while *G. vaginis* lacks these key enzymes. In the N_CE group, *L. crispatus* expressed high levels of *LacZ*, effectively degrading lactose, resulting in low levels of galactose. In the CE group, *G. vaginalis* proliferated excessively, which may inhibit the activity of lactobacilli or compete for substrates, leading to incomplete lactose breakdown. In addition, although *G. vaginalis* can express *LacZ* (as in some strains), its metabolic preference may be more inclined to utilize glucose rather than galactose; meanwhile, the dominant bacteria in the CE group, such as *Fannyhessea* and *Alloscardovia*, are mostly protein degrading bacteria, which may preferentially utilize amino acids rather than carbohydrates, further leading to the accumulation of galactose. This metabolic defect may be related to the pathogenic mechanism of *G. vaginalis*, as a pro-inflammatory metabolite, can exacerbate cervical epithelial inflammation by activating the host TLR signaling pathway (35), while the galactose degradation ability of *L. crispatus* plays a protective role (36). This could pave the way for early diagnosis, providing a new avenue for understanding the basis of preterm birth in older mothers.

Several studies have investigated the links between preterm birth and maternal health status, including diet (37), smoking (38), obesity (39), stress (40), age (41), and other demographic characteristics, such as socioeconomic, educational, and marital status (42). Schummers et al. (2018) found that women aged 35 or older at the time of delivery were associated with an increased risk of spontaneous preterm delivery and adverse fetal and infant outcomes, particularly at short interpregnancy intervals (43). Some studies have suggested that maternal gravidity is associated with an increased risk of adverse pregnancy outcomes in humans (44–46). Our results demonstrated that older mothers had higher gravidity than other pregnant women, ranging from 3 to 5. A previous study reported that even subtle differences in parity are associated with changes in the gut microbiome during pregnancy (47). However, whether gravidity affects the maternal vaginal microbiome during pregnancy remains unclear. This study found the presence of several genera, including *Lactobacillus, Gardnerella, Streptococcus, Alloscardovia, Fannyhessea, Sneathia, Klebsiella,* and *Achromobacter*. However, there was no significant difference between the G1-2 and G3-5 groups. These findings suggest that maternal gravidity may not be the main factor affecting preterm births in older mothers.

Bosdou et al. (2020) discovered that women undergoing *in vitro* fertilization-embryo transfer (IVF-ET) have an increased risk of GDM during early pregnancy (48). Furthermore, our findings indicate that older pregnant women had a significantly higher use of IVF-ET than other women, reaching 1/4. Koedooder et al. (49) demonstrated that multiple IVF processes may affect the vaginal microbiome (49). Therefore, it is reasonable to consider the impact of IVF-ET on the vaginal microbiome given the large number of unexplained preterm births. Moreover, prior evidence has shown that women with a low percentage of *Lactobacillus* spp. have a lower success rate for embryo implantation (50). In contrast, it is speculated that the use of IVF-ET may affect the composition of the vaginal microbiota in older mothers and, thus, affect pregnancy outcomes. This study observed that IVF-ET enriched the abundance of *Gardnerella, Alloscardovia*, and *Klebsiella*, emphasizing the need to consider assisted reproductive technologies in pregnancy studies; however, no significant difference was observed between them. These findings support the suggestion that IVF-ET may not interfere with preterm birth.

There is increasing evidence that cesarian delivery may have negative effects on maternal health (51) and increase the risk of obesity, type 2 diabetes (52), asthma (53), and other diseases (54) in the offspring. However, few studies have suggested that higher maternal age could be associated with a lower risk of adverse health outcomes in the offspring. This may be due to the older mothers have higher socioeconomic status and more parenting experience (55). Another possible reasons for such adverse outcomes might be due to the differences of elective cesarean section whether the planned vaginal delivery or emergency cesarean section (56). In our study, older mothers had a high percentage of cesarian deliveries (71%). This association may be partially related to age-related physiological changes, including the fact that vaginal dysbiosis occurs more frequently during pregnancy in older mothers (57, 58). Other factors, such as obstetric complications or clinical decision-making patterns, might also contribute to this outcome. However, Costello et al. (59) reported that the vaginal ecosystem was not strongly affected by the progression of gestation or the labor approach (59). In line with these findings, we also observed no significant alterations in the vaginal microbiota across the various delivery modes. This challenges the notion that cesarean delivery is a primary factor influencing preterm birth in older mothers. This suggests that mode of delivery may not be the primary maternal factor influencing preterm birth in older mothers.

Recent studies have suggested that CE, commonly known as “cervical erosion,” may increase the risk of infection by exposing the columnar epithelium to a potential infectious inoculum (60). Ongoing research is still uncovering the full understanding of this association (61). CE is seen frequently in pregnant women, and it may vary in response to hormonal fluctuations and the use of hormonal contraceptives (62). It has also been hypothesized that intrauterine infections leading to preterm birth may be secondary to the emergence of the pathogen from the vagina (63). One of these studies has demonstrated that cervical infection and inflammation may increase the risk of PTB during pregnancy (64). However, limited research has been conducted on the relationship between cervical ectopy and preterm births. Moreover, the impact of the vaginal microbiome on cervical ectopy and the development of preterm births in older mothers has not yet been explored. Previous clinical studies have established bacterial (i.e., *chlamydia*, *gonorrhea*, *mycoplasma*, etc.) as risk factors of preterm births (65). Another study also showed that endocervical infection with Ureaplasma spp. was significantly associated with preterm births (66). This study found that pregnant women with cervical ectopy had higher levels of *G. vaginalis* and lower levels of *L. crispatus*, *Bifidobacterium dentium*, and *Lactobacillus* iners. These findings suggest that CE may contribute to preterm birth in older mothers. The study sheds light on the potential role of *G. vaginalis* infection, and local inflammation in the cervix may compromise cervical integrity and result in premature remodeling, predisposing to preterm birth in older mothers.

*Gardnerella* species, such as *G. vaginalis*, have been identified as potential contributors to poor pregnancy outcomes, including bacterial vaginosis, sexually transmitted diseases, and preterm births (67). However, a clear consensus on the relationship between *G. vaginalis* and preterm births has not yet been established, and the specific mechanisms underlying *G. vaginalis* infection in preterm births are not well understood. *G. vaginalis* expresses several factors that enhance its virulence potential. These include adherence to CV epithelial cells, biofilm-forming capabilities, mucous degradation (sialidase) (68), and host-cell cytotoxicity (69). William et al. (2021) reported that *G. vaginalis* participates in choline metabolism, which can cause preterm birth (24). Liao et al. (26) proposed that *Gardnerella* species, which exhibit high intra-species genetic diversity, may contribute to preterm birth (26). This diversity could drive more frequent recombination events and stronger purifying selection in genes associated with lipid metabolism, potentially influencing host-pathogen interactions. Moreover, Fettweis et al. (12) suggested that women who experience preterm birth are more likely to have *G. vaginalis*, which is linked to a vaginal cytokine profile richer in pro-inflammatory cytokines, such as exotoxin, IL-1β, IL-6, and MIP-1β (44). Anton et al. suggested that *G. vaginalis* significantly increases epithelial cell death and decreases epithelial barrier integrity. This can regulate the NF-κB pathway through the TLR2 signaling pathway, promoting cytokine release and the epithelial immune response, ultimately leading to adverse pregnancy outcomes (12). These results support our findings regarding differences in the vaginal microbiome. *G. vaginlis* was associated with CE, suggesting that it is a significant contributor to maternal premature birth. *G. vaginalis* showed a positive correlation with D-galactose, which has been linked to premature ovarian failure and subacute aging *in vivo* (70). Elevated D-galactose levels mediated by *Gardnerella vaginalis* were significantly associated with maternal preterm birth; particularly in older mothers with CE, this association may be attributed to the reduced abundance of *Lactobacillus*, which normally metabolize galactose.

Feehily et al. (19) confirmed the association of a vaginal microbiome dominated by *L. crispatus* with full-term pregnancies by using a shotgun metagenomic approach (19). Roux et al. (1993) also suggested that *L. crispatus* plays a key role in protecting against preterm birth (71). Furthermore, *L. crispatus* has been linked to the stability of vaginal microbiota during pregnancy (72). Consistent with these findings, we observed a lower presence of *L. crispatus* in older mothers with CE than in those without. These findings suggest an antagonistic relationship between *the Lactobacillus* species and premature birth. However, *L. iners* have been shown to promote premature birth in a similar cohort (17). In contrast to previous reports, we did not observe an increase in *L. iners*, which is associated with vaginosis (73). Furthermore, the mechanistic basis of this role remains unclear. Voltan et al. suggested that *L. crispatus* prevents premature birth by decreasing inflammation through H2O2 signaling to control NF-κB activity (74). Moreover, it can inhibit bacteria associated with bacterial vaginitis (75). This study presents evidence of a previously unknown mechanism linking the vaginal microbiota and galactose levels in the vagina. Women with galactosemia, an inborn metabolic error in which transferases are absent, usually experience premature ovarian failure and infertility (76). *Bifidobacteria* are believed to be the primary organisms involved in lactose and related β-galacto-oligosaccharides metabolism (77). Studies have indicated that the Leloir pathway is a common galactose metabolism pathway in lactic acid bacteria, and the source of intracellular galactose is usually transport protein-mediated galactose input or beta-galactosidase (3.2.1.23) hydrolysis of lactose in cells (derived from transport protein-mediated transport). After entering the Leloir pathway, beta-galactose is first converted to alpha-D-galactose by the catalysis of galactose mutarotase (5.1.3.3) (78). In this study, we found an inverse correlation between galactose and *L. crispatus* abundance. This species possesses genes that encode for D-galactonate degradation through the DD pathway (D-galactonate => glycerate-3P) and the Leloir pathway (galactose => alpha-D-glucose-1P) (M00552 and M00632, respectively). Meanwhile, we discovered that GH2 genes, which hydrolyze lactose commonly found in Firmicutes, participate in the “galactose metabolism” pathway and are enriched in the genome of *L. crispatus*. Therefore, the relationship between the enzyme activity of *L. crispatus* and galactose levels in the serum and vagina should be investigated in future studies. It has been hypothesized that bacteria in the vagina that express D-galactonate degradation enzymes may share the characteristic of reducing galactose, which could lead to a decrease in the incidence of preterm birth.

This study had a few limitations. First, this study has its small sample size; therefore, further large-scale studies are needed to validate the findings and establish causality. Our results cannot conclusively determine causality between *G. vaginalis* dysbiosis and CE, D-galactose and CE, or CE and preterm birth. These relationships should be confirmed in germ-free mice and in vaginal microbiota transplantation (VMT). Furthermore, our results indicate that galactosidase is more commonly distributed in *L. crispatus* in older mothers with non-CE. Further studies should investigate the use of engineered *E. coli* expressing galactosidase in the vagina to prevent preterm births. Moreover, exploring how the vaginal microbiome modulates metabolic pathways in older mothers with cervical ectopy is of interest. The potential clinical applications of the study, such as early diagnosis using machine learning models, warrant further exploration and validation in diverse populations.

In conclusion, this study found that age range categories, i.e., 17–22 year old and 36–41 year old mothers were at a higher risk of premature birth. Factors such as maternal gravidity, IVF-ET use, birth delivery mode, and CE may influence the occurrence of premature birth. Furthermore, older mothers with CE exhibited vaginal microbiome dysbiosis, including an increase in *G. vaginalis* and a reduction in *L. crispatus*. Disturbances in the vaginal microbiome may contribute to CE by affecting carbohydrate metabolism, such as galactose metabolism, in the host. Associations between specific bacterial genera (e.g., *Gardnerella*, *Lactobacillus*) and metabolites (e.g., D-galactose) with cervical ectopy (CE) and preterm birth are identified. Specific species and metabolites of the vaginal microbiota were identified as equally effective in the early detection of premature birth, providing a non-invasive screening method for premature birth in older mothers with CE.

## MATERIALS AND METHODS

### Subject recruitment and sample collection

The study recruited pregnant individuals of varying ages from The Sixth Affiliated Hospital, Sun Yat-sen University, from January 2020 to December 2022. The study protocol was approved by the Ethics Committee of the Sixth Affiliated Hospital of Sun Yat-sen University (No.2021ZSLYEC-513), and all participants provided written informed consent before sample collection.

All participants were local residents of South China with no history of smoking or alcohol consumption. None of the participants had received any drugs, antibiotics, probiotics, or prebiotics within 1 month before sampling. The clinical information of all pregnant women was recorded, including age, height, weight, BMI, gestational age, gravidity, parity, systolic pressure (SBP), and diastolic pressure (DBP), as well as any adverse pregnancy outcomes, such as fetal growth restriction, low birth weight infants, and premature birth. This study discusses the use of fertilization-embryo transfer (IVF-ET) and pregnancy comorbidities, including columnar ectopy, scarred uterus, uterine myoma, uterine adenomyosis, amniotic fluid, oligohydramnios, polyhydramnios, pelvic infection, polycystic ovary syndrome (PCOS), intrahepatic cholestasis of pregnancy (ICP), gestational diabetes mellitus (GDM), obesity, preeclampsia, anemia, group B streptococcus (GBS) infection, cardiovascular (CV) infection, and *Escherichia coli* infection. This study investigated various factors that could contribute to complications during childbirth, including *Escherichia coli* infection, premature rupture of membranes (PROM), fetal distress, perineal laceration, gluteal leakage, uterocervical angle notch (UCAN), placental adhesions, and delivery mode (cesarian, vaginal birth, and abortion). Vaginal swabs were collected and stored at −80°C until further analysis.

### Clinical laboratory tests

Biochemical indicators of vaginal discharge from pregnant women were measured using an enzyme-linked immunosorbent assay (ELISA) kit (Beijing Winter Song Boye Biotechnology Co., Ltd., Beijing, China). The indicators measured included Interleukin (IL-1β), IL-6, IL-8, IL-10, human chorionic gonadotropin (HCG), progesterone (PROG), estrogen (E), Hydroxybutyrate dehydrogenase (HBD-2), G protein-coupled receptor 30 (GPR30), and C-reactive protein (CRP) and were measured according to the manufacturer’s instructions.

### DNA extraction, PCR amplification, and sequencing

DNA was extracted as previously described (79). The E.Z.N.A. soil DNA Kit (Omega Bio-tek, Norcross, GA, USA) was used to extract DNA from vaginal discharge samples according to the manufacturer’s protocol. Paired-end sequencing was performed on an Illumina NovaSeq/Hiseq Xten (Illumina Inc., San Diego, CA, USA) using the NovaSeq Reagent Kits/HiSeq X Reagent Kits (https://www.illumina.com/).

### 16S rRNA gene data processing

Then, the high-quality sequences were de-noised using DADA2 (80) plugin in the QIIME2 (81) (version 2020.2) pipeline with recommended parameters, which obtains single-nucleotide resolution based on error profiles within samples. DADA2 denoized sequences are usually called amplicon sequence variants (ASVs). To minimize the effects of sequencing depth on alpha and beta diversity measure, the number of sequence from each sample was rarefied to 4,000, which still yielded an average Good’s coverage of 97.90%. Taxonomic assignment of ASVs was performed using the Vsearch consensus taxonomy classifier implemented in QIIME2 and the SILVA 16S rRNA database (v138). Analyses of the 16S rRNA microbiome sequencing data were performed using the free online platform of Majorbio Cloud Platform (https://www.majorbio.com/). The raw reads were deposited into the NCBI Sequence Read Archive (SRA) database (Accession Number: PRJNA1289226).

### Metagenomic data processing

The data were analyzed on the free online platform of Majorbio Cloud Platform (https://www.majorbio.com/). Briefly, the paired-end Illumina reads were trimmed of adaptors, and low-quality reads (length <50 bp or with a quality value <20 or having N bases) were removed by fastp (82). Reads were aligned to the human genome by BWA (83) (http://bio-bwa.sourceforge.net, version 0.7.9a) and any hit associated with the reads and their mated reads were removed. Metagenomics data were assembled using MEGAHIT (84), which makes use of succinct de Bruijn graphs. Contigs with with a length ≥300 bp were selected as the final assembling result, and then, the contigs were used for further gene prediction and annotation. Non-redundant gene catalog were aligned to NR database with an e-value cutoff of 1e^−5^ using Diamond (83) for taxonomic annotations. The KEGG annotation was conducted using Diamond (85) against the Kyoto Encyclopedia of Genes and Genomes database with an *e*-value cutoff of 1e^−5^. Carbohydrate-active enzyme annotation was performed in the CAZy database using hmmScan.

### Vaginal metabolome analysis

To extract metabolites, the vaginal swab was immersed in saline and mixed evenly using a vortex apparatus (86). The mixture was then centrifuged, and the supernatant was collected. The supernatant (100 µL) was suspended in 300 µL of a solvent composed of methanol and acetonitrile at a 2:1 ratio (vol:vol). The mixture was vortexed for 1 min and stored at −20°C for 2 h. Finally, the mixture at 10,000 rpm for 20 min at 4°C. The liquid remaining after centrifugation was concentrated in a cryovacuum and 150 µL of a complex solution (methanol: H_2_O = 1:1, vol:vol) was added for resolution. The mixture was vortexed for 1 min and centrifuged at 10,000 rpm for 30 min at 4°C. The resulting supernatant was collected and placed in a sample bottle for liquid chromatography-mass spectrometry (LC-MS/MS) analysis. Ten microliters of supernatant from each sample was mixed with the QC samples to evaluate the repeatability and stability of the LC-MS analysis process.

The LC-MS/MS collected raw data were processed using Compound Discoverer 3.1 (Thermo Fisher Scientific, USA). Processing included peak extraction, intra- and inter-group retention time correction, adjoint ion merging, missing value filling, background peak labeling, and metabolite identification. The molecular weights, retention times, peak areas, and identification results for the compounds were determined. The metabolites were identified by combining several databases, including the BGI Library, mzCloud, and ChemSpider (HMDB, KEGG, and LipidMaps).

### Complete genome sequencing and β-galactosidase activity determining in *Lactobacillus crispatus*

*L. crispatus* G654-8 was isolated from vaginal swab samples. The genome of G654-8 was sequenced using the PacBio Sequel and Illumina HiSeq 4000 platforms (BGI, Shenzhen, China). Single-base correction was performed using Cutadapt (v1.9.1) and single-molecule real-time (SMRT) to improve the genome sequencing accuracy. The filtered Illumina reads were then mapped to a bacterial plasmid database using SOAP to identify plasmids. Functional annotation was performed using the BLAST (version 2.2.31+) alignment tool. The KEGG database was used for functional annotation. Beta-galactosidase activity of *L. crispatus* G654-8 was determined by hydrolyzing 5-bromo-4-chloro-3-indoyl-beta-d-galactopyranoside (Xgal) on tryptic soy agar plates (87). Secondary and tertiary structures of the β-galactosidase protein were constructed using the GOR IV secondary structure prediction method (https://npsa-prabi.ibcp.fr/cgi-bin/npsa) and SWISS-MODEL (https://swissmodel.expasy.org/interactive), respectively.

### Statistical analysis

Statistical analyses were conducted using SPSS 22.0 (SPSS Inc., Chicago, IL, USA). Mean ± standard deviation (SD) represents quantitative data, whereas frequency and percentage represent count data. To compare the percentages between two or more groups, a *χ*2 test was used. One-way analysis of variance was conducted using the nonparametric Kruskal–Wallis test. Multiple tests between multiple groups were performed using Dunnett’s multiple comparisons test. Statistical significance was set at *P <* 0.05. Statistical analyses were performed using GraphPad Prism 8.0 software (GraphPad Software, USA). MetaboAnalyst 5.0 was used for non-targeted metabolomics. The linear discriminant analysis (LDA) effect size (LEfSe) module of the Galaxy/HutLab platform was used to analyze differential intestinal microbial genera between groups. The correlation of clinical indicators with vaginal microbiota and metabolites was analyzed using the pheatmap package in R (ver. 4.3.2).

## Data Availability

The raw reads were deposited into the NCBI Sequence Read Archive (SRA) database (Accession Number: PRJNA1289226).

## SUPPLEMENTAL MATERIAL

Data S1 msystems.00825-25-s0001.xlsx *L. crispatus* may contribute to the lower circulating concentrations of galactose in subjects with CE.

Fig. S1 msystems.00825-25-s0002.tif Clinical characteristics and biochemical indicators among parturients of different reproductive ages.

Fig. S2 msystems.00825-25-s0003.tif Alpha-diversity and beta-diversity of vaginal microbial community of among parturients of different reproductive ages.

Fig. S3 msystems.00825-25-s0004.tif

Fig. S4 msystems.00825-25-s0005.tif Alpha-diversity, beta-diversity, and composition of vaginal microbial community in the older mothers with different numbers of gravidity.

Fig. S5 msystems.00825-25-s0006.tif Alpha-diversity, beta-diversity, and composition of vaginal microbial community in the older mothers with using IVF-ET.

Fig. S6 msystems.00825-25-s0007.tif Alpha-diversity, beta-diversity, and composition of vaginal microbial community in the older mothers with selection of different delivery methods.

Fig. S7 msystems.00825-25-s0008.tif Comparison of the relative abundance of vaginal microbes in the older mothers with columnar ectopy and non-columnar ectopy.

Fig. S8 msystems.00825-25-s0009.tif Enzymes related to galactose metabolism (ko00052) pathways were annotated.

Supplemental legends msystems.00825-25-s0010.docx Legends for supplemental files.

ASM does not own the copyrights to Supplemental Material that may be linked to, or accessed through, an article. The authors have granted ASM a non-exclusive, world-wide license to publish the Supplemental Material files. Please contact the corresponding author directly for reuse.
