# Supplementary figures and images for: Increased vaginal Gardnerella vaginalis abundance and reduced D-galactose metabolism are associated with preterm birth in older mothers with columnar ectopy in South China
Source: mSystems. 2025 Aug 15;10(9):e00825-25. doi: 10.1128/msystems.00825-25 (PMC12455998; doi:10.1128/msystems.00825-25)

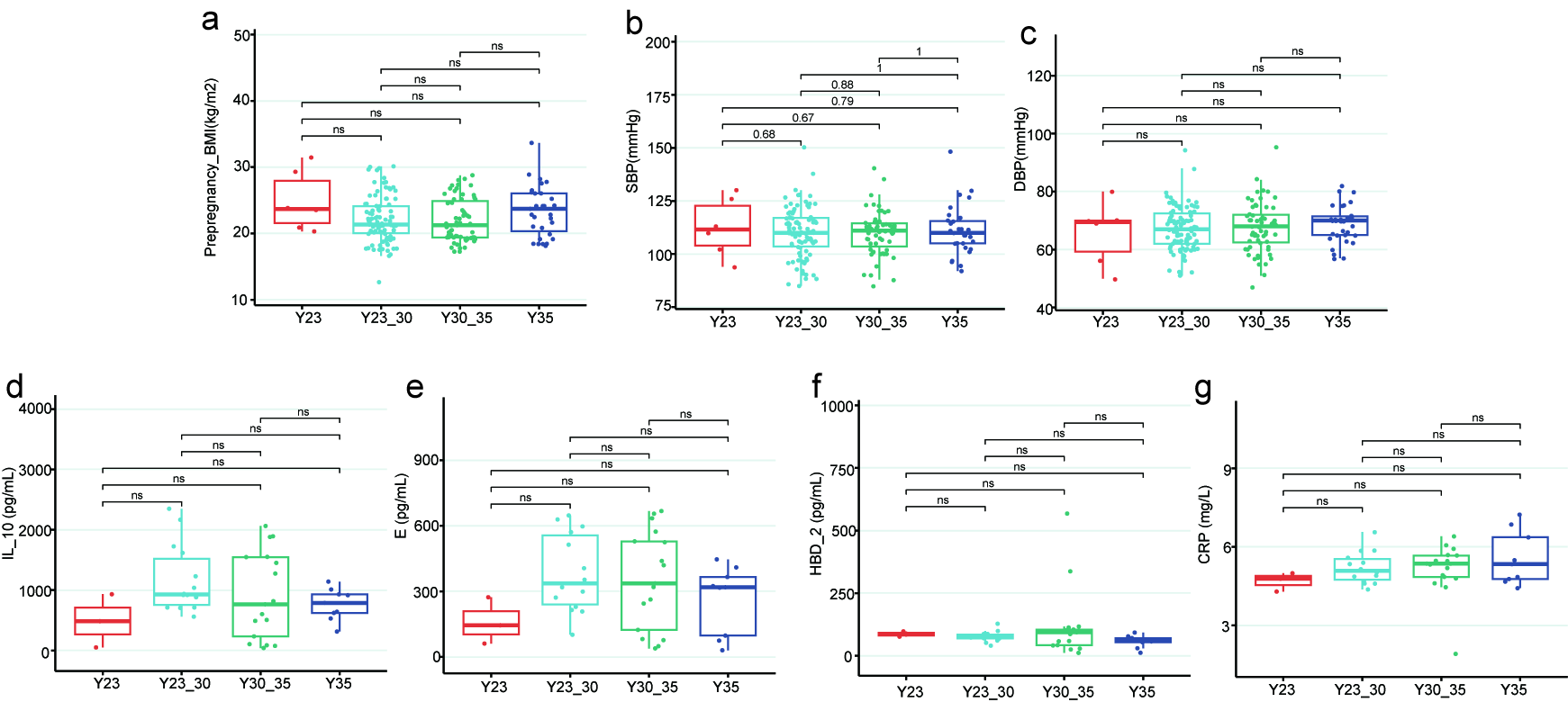

Supplement: Fig. S1 — Clinical characteristics and biochemical indicators among parturients of different reproductive ages. [file msystems.00825-25-s0002.tif]

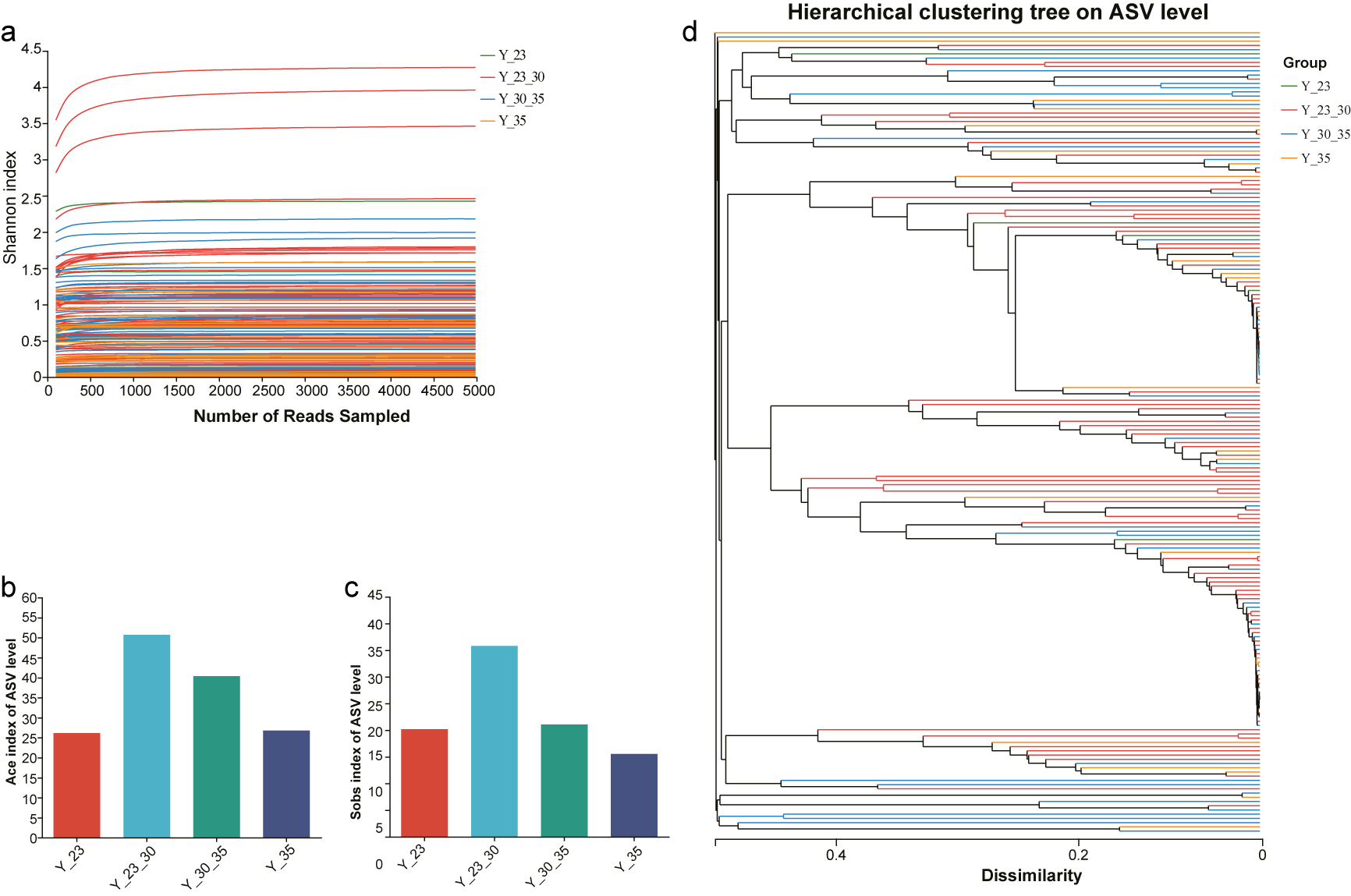

Supplement: Fig. S2 — Alpha-diversity and beta-diversity of vaginal microbial community of among parturients of different reproductive ages. [file msystems.00825-25-s0003.tif]

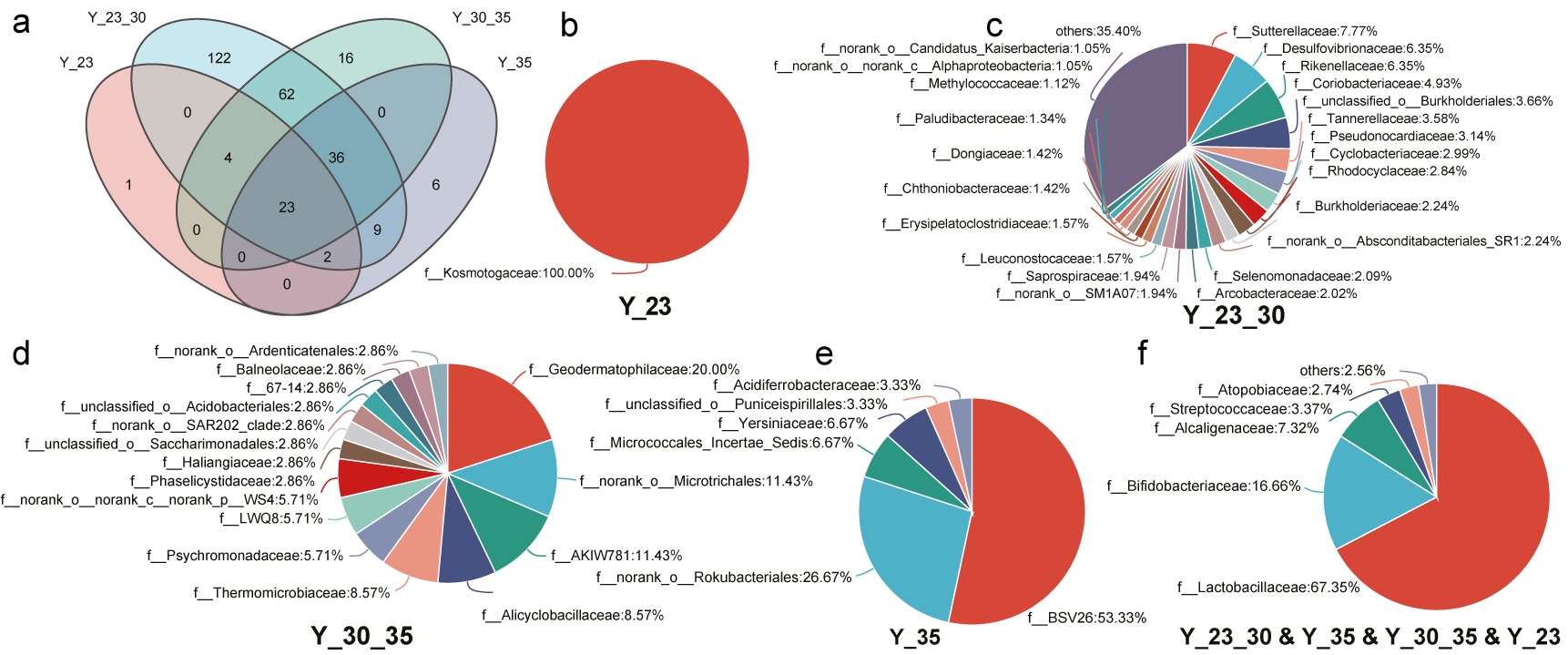

Supplement: Fig. S3 [file msystems.00825-25-s0004.tif]

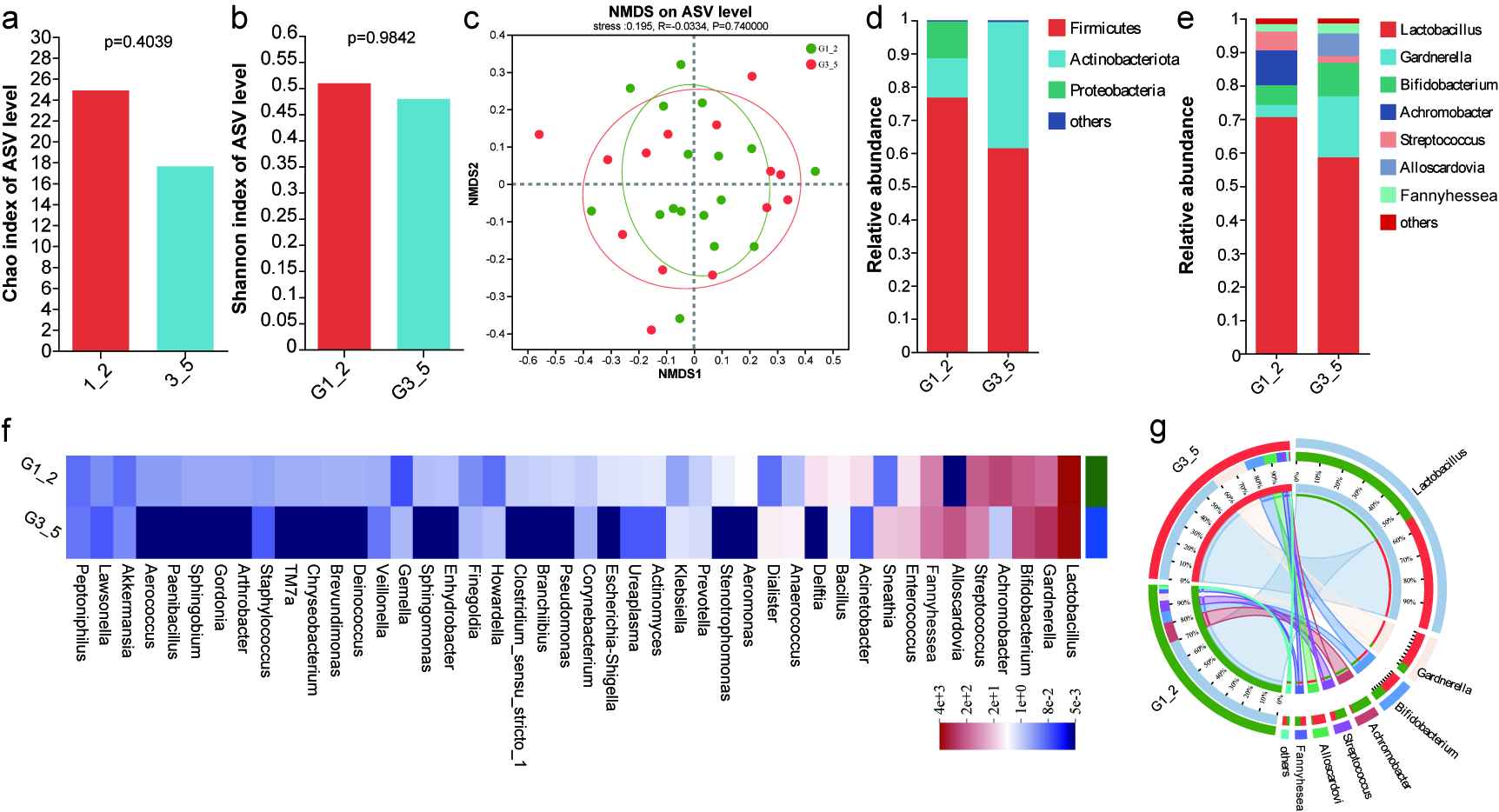

Supplement: Fig. S4 — Alpha-diversity, beta-diversity, and composition of vaginal microbial community in the older mothers with different numbers of gravidity. [file msystems.00825-25-s0005.tif]

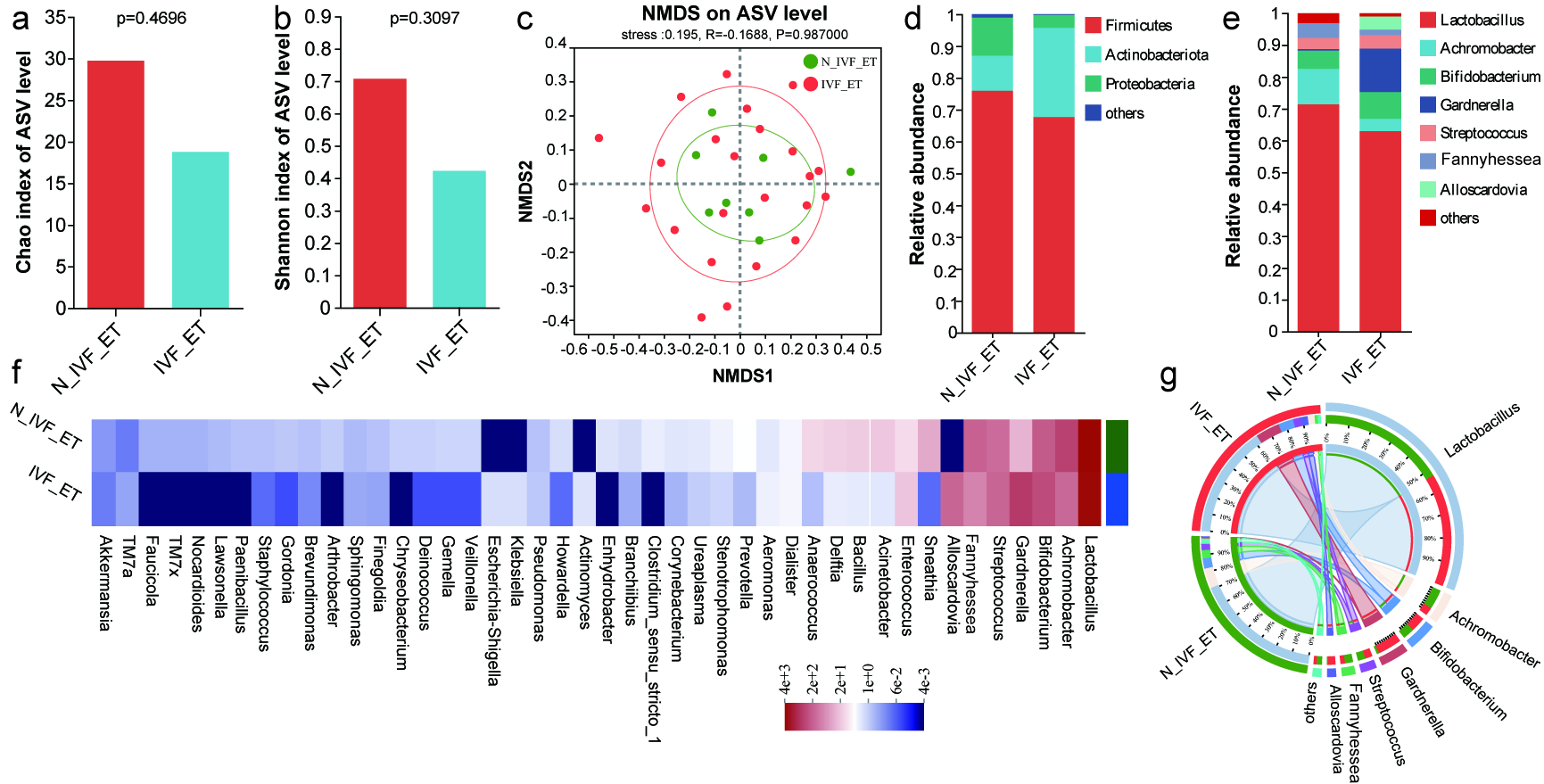

Supplement: Fig. S5 — Alpha-diversity, beta-diversity, and composition of vaginal microbial community in the older mothers with using IVF-ET. [file msystems.00825-25-s0006.tif]

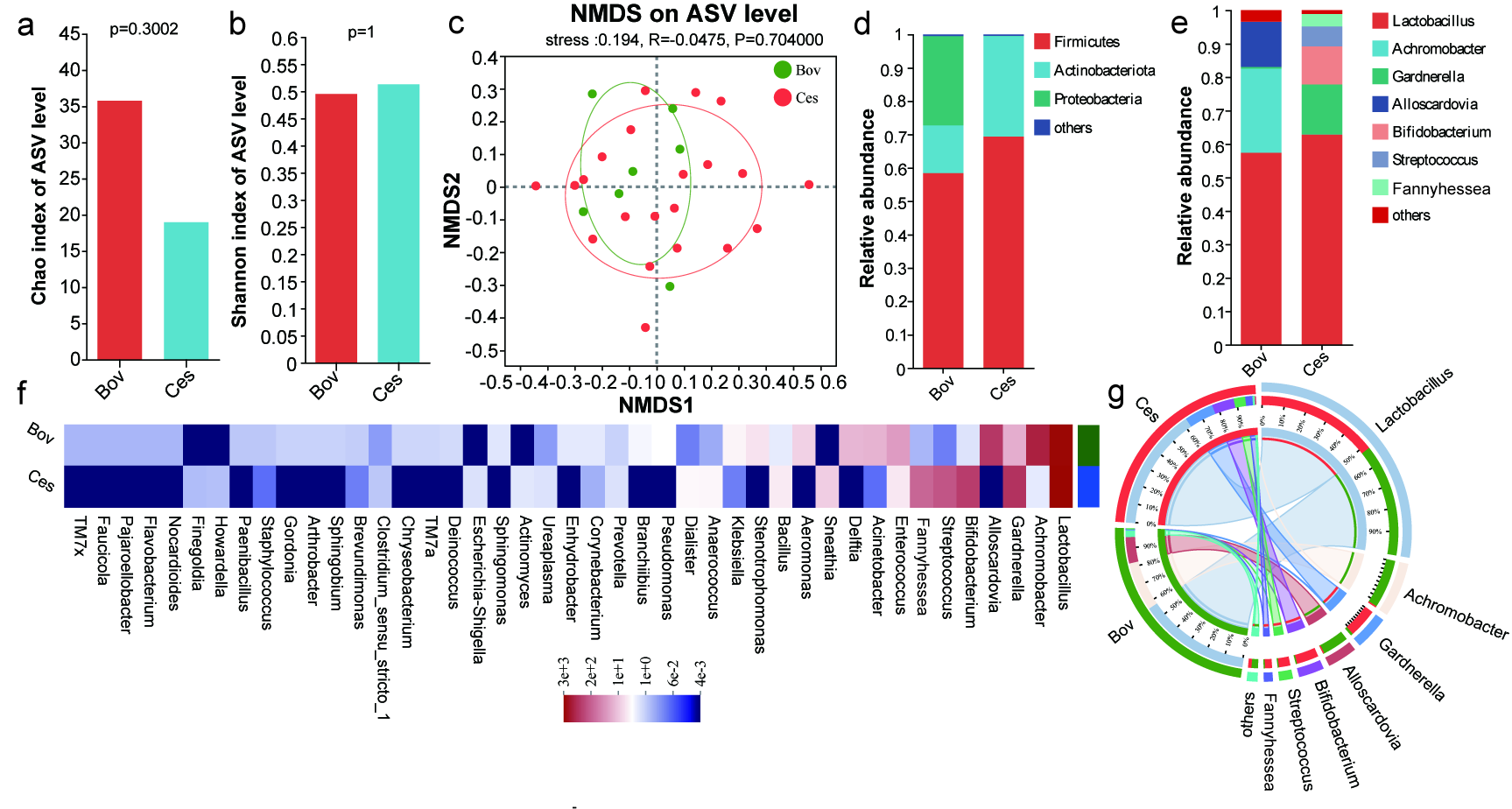

Supplement: Fig. S6 — Alpha-diversity, beta-diversity, and composition of vaginal microbial community in the older mothers with selection of different delivery methods. [file msystems.00825-25-s0007.tif]

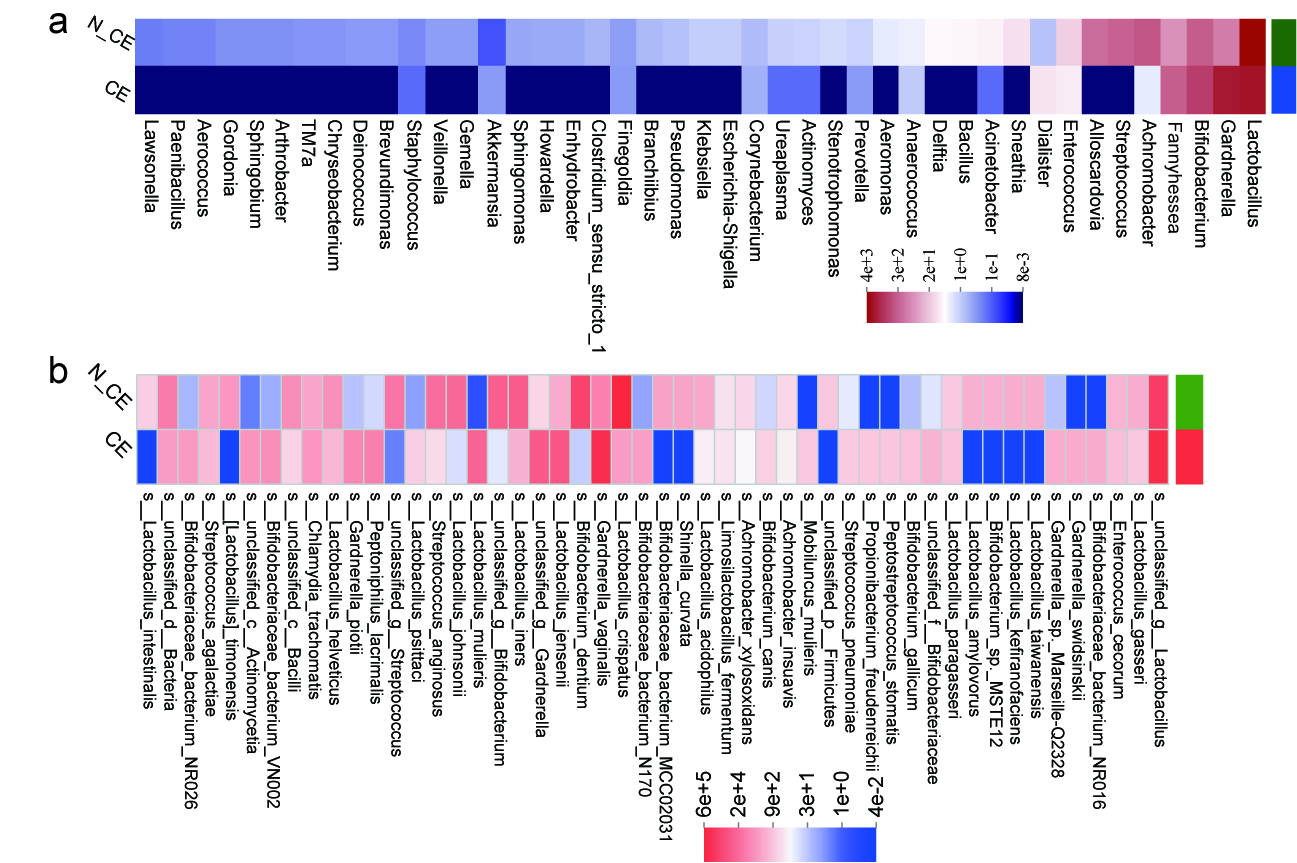

Supplement: Fig. S7 — Comparison of the relative abundance of vaginal microbes in the older mothers with columnar ectopy and non-columnar ectopy. [file msystems.00825-25-s0008.tif]

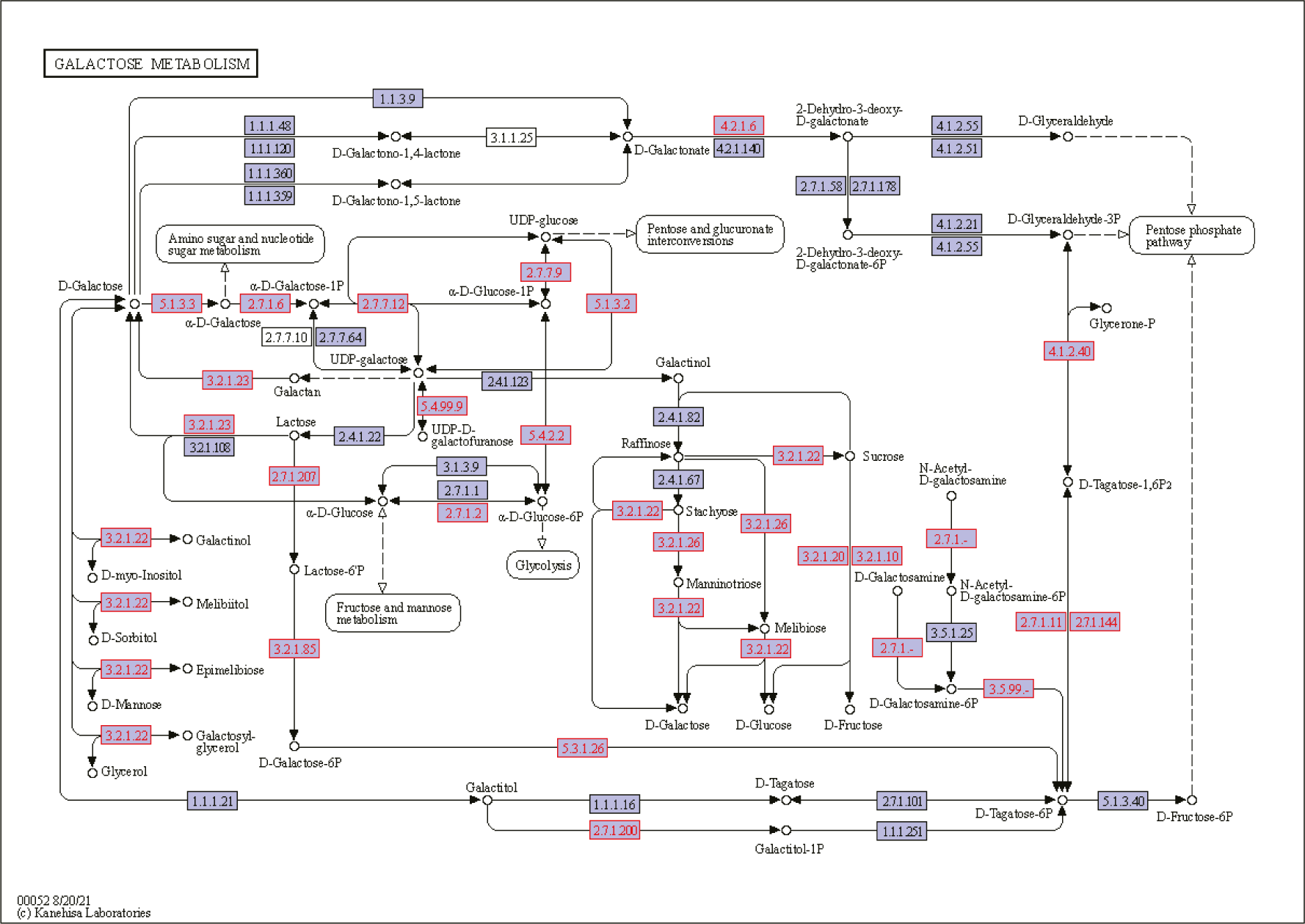

Supplement: Fig. S8 — Enzymes related to galactose metabolism (ko00052) pathways were annotated. [file msystems.00825-25-s0009.tif]
