# Supplementary material for: Increased vaginal Gardnerella vaginalis abundance and reduced D-galactose metabolism are associated with preterm birth in older mothers with columnar ectopy in South China
Source: mSystems. 2025 Aug 15;10(9):e00825-25. doi: 10.1128/msystems.00825-25 (PMC12455998; doi:10.1128/msystems.00825-25)
Supplement: Supplemental legends — Legends for supplemental files. [file msystems.00825-25-s0010.docx]

**Data S1.** *L. crispatus* may contribute to the lower circulating concentrations of galactose in subjects with CE.

**Figure captions**

**Fig. S1.** Clinical characteristics and biochemical indicators among parturients of different reproductive ages. (a) Prepregnancy_BMI. (b) SBP. (c) DBP. (d) IL-10. (e) E. (f) HBD-2. (g) CRP. BMI: body mass index; SBP: systolic pressure; DBP: diastolic pressure; IL-10: interleukin-10; E: estrogen; HBD-2: human beta-defensin-2; CRP: C-reactive protein.

**Fig. S2.** Alpha-diversity, beta-diversity of vaginal microbial community of among parturients of different reproductive ages. (a) Shannon curve for each sample. (b) The microbial diversity estimated by Ace index. (c) The microbial diversity estimated by Sobs index. (d) Hierarchical clustering tree of the vaginal bacterial community in parturients of different reproductive ages on ASV level.

**Fig. S3.** Venn diagrams and pieplot of the parturients of different reproductive ages according to vaginal microbial biodiversity at the family level. (a) Venn diagrams of the parturients of different reproductive ages according to vaginal microbial biodiversity at the family level. (b) The pieplot of unique vaginal microbial community in teenager mothers at the family level. (c) The pieplot of vaginal microbial community in Y23-30 mothers at the family level. (d) The pieplot of unique vaginal microbial community in Y30-35 mothers at the family level. (e) The pieplot of unique vaginal microbial community in older mothers at the family level. (f) The pieplot of common vaginal microbial community in parturients of different reproductive ages at the genus level.

**Fig. S4.** Alpha-diversity, beta-diversity and composition of vaginal microbial community in the older mothers with different number of gravidity. (a) The microbial diversity estimated by Chao index. (b) The microbial diversity estimated by Shannon index. (c) NMDS analysis of the bacterial community in the older mothers with different number of gravidity at the ASV level. (d) Relative abundance of the microbial community at the phylum level in the older mothers with different number of gravidity. (e) Relative abundance of the microbial community at the genus level in the older mothers with different number of gravidity. (f) Heatmap of the microbial community at the genus level in the older mothers with different number of gravidity. (g) Circos of the microbial community at the genus level in the older mothers with different number of gravidity. (h) Comparison of the relative abundance of vaginal microbes in the older mothers with different number of gravidity. ^*^*p* < 0.05, ^**^*p* < 0.01, ^***^*p* < 0.001.

**Fig. S5.** Alpha-diversity, beta-diversity and composition of vaginal microbial community in the older mothers with using IVF_ET. (a) The microbial diversity estimated by Chao index. (b) The microbial diversity estimated by Shannon index. (c) NMDS analysis of the bacterial community in the older mothers with using IVF_ET at the ASV level. (d) Relative abundance of the microbial community at the phylum level in the older mothers with using IVF_ET. (e) Relative abundance of the microbial community at the genus level in the older mothers with using IVF_ET. (f) Heatmap of the microbial community at the genus level in the older mothers with using IVF_ET. (g) Circos of the microbial community at the genus level in the older mothers with using IVF_ET. (h) Comparison of the relative abundance of vaginal microbes in the older mothers with using IVF_ET. ^*^*p* < 0.05, ^**^*p* < 0.01, ^***^*p* < 0.001.

**Fig. S6.** Alpha-diversity, beta-diversity and composition of vaginal microbial community in the older mothers with selection of different delivery methods. (a) The microbial diversity estimated by Chao index. (b) The microbial diversity estimated by Shannon index. (c) NMDS analysis of the bacterial community in the older mothers with selection of different delivery methods at the ASV level. (d) Relative abundance of the microbial community at the phylum level in the older mothers with selection of different delivery methods. (e) Relative abundance of the microbial community at the genus level in the older mothers with selection of different delivery methods. (f) Heatmap of the microbial community at the genus level in the older mothers with selection of different delivery methods. (g) Circos of the microbial community at the genus level in the older mothers with selection of different delivery methods. (h) Comparison of the relative abundance of vaginal microbes in the older mothers with selection of different delivery methods. ^*^*p* < 0.05, ^**^*p* < 0.01, ^***^*p* < 0.001.

**Fig. S7.** Comparison of the relative abundance of vaginal microbes in the older mothers with columnar ectopy and non_columnar ectopy. (a) Comparison of the specific vaginal microbes in the older mothers with columnar ectopy and non_columnar ectopy. (b) Heatmap of the microbial community at the genus level in the older mothers with columnar ectopy and non_columnar ectopy. (c) Heatmap of the microbial community at the species level in the older mothers with columnar ectopy and non_columnar ectopy.

**Fig. S8.** Enzymes related to galactose metabolism (ko00052) pathways were annotated.
